# Ultrasound-Assisted Synthesis of 2‑Benzylidene-1-Indanone Derivatives and Evaluation as a Corrosion Inhibitor for Mild Steel in 1 M HCl Solution

**DOI:** 10.1021/acsomega.4c09705

**Published:** 2025-05-20

**Authors:** Ricardo Ballinas-Indili, Paola Roncagliolo-Barrera, Roberto Salcedo, Francisco J. Rodríguez-Gómez, Cecilio Álvarez-Toledano

**Affiliations:** † Departamento de Ciencias Químicas, Facultad de Estudios Superiores Cuautitlán Campo 1, Universidad Nacional Autónoma de México, Estado de México 54740, México; ‡ Departamento de Ingeniería Metalúrgica, Facultad de Química, Ciudad Universitaria, Ciudad de México 04510, México; § Instituto de Investigaciones en Materiales, Universidad Nacional Autónoma de México, Ciudad Universitaria, Ciudad de México 04510, México; ∥ Instituto de Química, Universidad Nacional Autónoma de México, Ciudad Universitaria, Ciudad de México 04510, México

## Abstract

This study explores the ultrasound-assisted synthesis of three 2-benzylidene-1-indanone derivatives and assesses their effectiveness as corrosion inhibitors for mild steel AISI 1018 in 1 M HCl through electrochemical techniques and density functional theory (DFT) quantum chemical calculations. The results indicate that the inhibitors studied; specifically IND-1, (Z)-2-(hydroxy­(phenyl)­methylene)-2,3-dihydro-1H-inden-1-one; IND-2, (Z)-2-(hydroxy­(pyridine-3-yl)­methylene)-2,3-dihydro-1H-inden-1-one and IND-3, (Z)-2-((4-(dimethylamino)­phenyl)­(hydroxy)­methylene)-2,3-dihydro-1H-inden-1-one, modified the cathodic reaction mechanism. These inhibitors achieve efficiencies greater than 98%, and their inhibitory effect does not increase with higher concentrations. A comparison of computational predictions and experimental analyses indicates that the pyridine ring and the diethylamino group reduce the inhibitory capacity of carbon steel in acidic environments.

## Graphical abstract

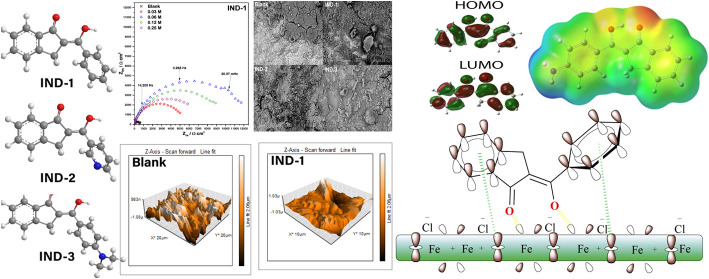

## 1 Introduction

Inhibitors effectively minimize damage to metals, making them a cost-effective method of protecting steel from corrosion in closed systems. Due to their preventive and protective qualities, this method is successfully used in industrial applications that favor sustainable development and conservation of natural resources.^1–3^

Organic compounds are often used for their adsorption mechanism, which provides uniform and durable coverage, even in hard-to-reach areas. Numerous studies have described the advantages offered by compounds with heteroatoms, such as nitrogen (N), oxygen (O), sulfur (S), and phosphorus (P), possessing free electron pairs that can form coordination bonds with the empty *d orbitals* of the iron in steel.^4–7^ Scientific literature has found that molecules containing N atoms achieve high yields due to the bonding between free electron pairs and empty orbitals of metal atoms. Still, it has been shown that for molecules containing extensive π-systems, such as aromatic rings, these *π-d* interactions facilitate adsorption and form a more robust and stable protective film. They have a larger surface area to interact with the metal surface. Aromaticity is another factor, as electrons function as chelators to form multiple bonds on the metal surface, especially under acidic conditions.^8–12^ When designing and evaluating new organic compounds, it is imperative to consider these effects.

Indanones are polycyclic ketones with a benzene ring attached to cyclopentanone rings and present fascinating chemical structures and physical properties, which have aroused great interest in scientific research from structural designs and new sustainable synthesis methods. Numerous scientific studies have confirmed its multiple beneficial properties, including its antibacterial, antispasmodic, and anti-inflammatory effects. In addition to their therapeutic potential, they have shown promise in treating Alzheimer’s disease and could play an essential role in chemotherapy, especially in the fight against resistant cancer cells.^13–16^ They also have applications in materials science, for example, in electronic systems such as OLEDs (organic light-emitting diodes) and photochemical devices.^17–19^ Another significant advantage is the low toxicity compared to other conventional compounds containing phosphates, chromates, and heavy metals, the use of which is restricted. Research has shown that indanone is poorly toxic orally in mice, even at a dose of 1000 mg/kg, with no appreciable adverse effects on blood clearance, absorption time, or half-life.^20–23^

The effect of tautomeric compounds has been extensively studied in corrosion inhibitors, focusing on amine compounds from ketones and contrasting them with aniline-derived compounds. NH compounds show tautomeric isomers related to the Schiff base, differentiated by their keto–enol forms. These forms influence molecular interactions with metal surfaces and enhance corrosion protection via enol-keto and diketo interactions of 1,3-diketone malonates.^24–27^

A key advantage of this compound is its structural diversity and ability to undergo enol-ketone tautomerism, mainly due to the essential hydroxyl group. Due to enol-keto tautomerism, indanones could change their molecular structure and become asymmetrical diketones. Theoretically, there are three different molecular configurations (see Scheme 1). Experiments show that only enol-keto form 1 and keto–keto form exist, with enol-keto being more stable due to its significant π-conjugation and a higher ratio than the keto–keto form.^28,29^

**Scheme 1:**
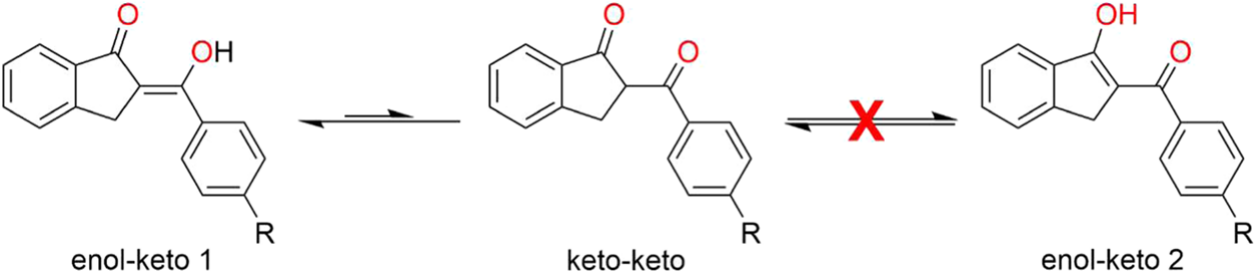
Tautomers of the 1,3-Diketone Derivative Indanone

In addition, they also proved to be metal chelators, a crucial anticancer property; fundamental structure–activity relationship analysis indicated that the base of a 1-indanone skeleton and the aryl group were coplanar and had been responsible for the selective cytotoxic response.^30–33^

Other indanone derivatives, evaluated as corrosion inhibitors, show 87–90% efficiency on metal surfaces via the 1,3-diacetone structure, enhancing adsorption and redox reactions. A. Saady et al. studied three modified indanone compounds, achieving up to 92% efficiency with a methyl group.^34^ Further research has developed a method for the preparation of b-hydroxybenzylidene-1-indanone derivatives characterized by a 1,3-diacetone structure, which is an essential class of 1,3-dicarbonyl compounds with strong metal chelating properties, which are chemically stable and generally nontoxic.^35–38^

Indanone derivatives’ design considers substituents affecting surface coverage and the orientation of organic corrosion inhibitors on metals. Research shows that polar electron-donating substituents improve heterocyclic corrosion inhibitors’ performance, making incorporation beneficial and preferable. The amino and diethylamino groups are versatile candidates for corrosion inhibition, enhancing the N-chain and improving inhibition efficiency by increasing the electron-donating capacity.^39,40^ Further study investigations have developed a method for the preparation of β-hydroxybenzylidene-1-indanone derivatives characterized by a 1,3-diacetone structure, which is an essential class of 1,3-dicarbonyl compounds with strong metal chelating properties, which are chemically stable and usually nontoxic.^41,42^ According to cyclic voltammetry tests, 1- and 2-indanones exhibit strong adsorption, demonstrating their capability to undergo chemisorption processes on a metal surface, mainly when the structural motif is 1,3-diketone.^43–46^

Standard methods for obtaining indanone derivatives include the Nazarov, Knoevenagel, and Diels–Alder reactions and Friedel–Crafts alkylation and acylation reactions.^47^ Our research uses an ultrasound-assisted synthesis method to improve the reaction yield that aligns with sustainability and green synthesis principles.^48^ During the synthesis process, the aromatic ring structures of three 2-benzylidene-1-indanone derivatives (IND-1, IND-2, and IND-3) were modified, and the inhibition efficiency of AISI 1018 carbon steel in hydrochloric acid (HCl) was evaluated. This modification aimed to clarify the adsorption mechanisms of indanone’s functional groups as a corrosion inhibitor.

## 2 Experimental Details

### 2.1 Synthesis of Hydroxybenzylidene-1-Indanone

The initial reagents, *o-*phthaldialdehyde (98%), acetophenone (99%), 3-acetylpyridine (98%), and 1-(4-(dimethylamino) phenyl) ethanone (99%), HCl (37% ACS reagent) and sodium hydroxide and were purchased from Sigma-Aldrich and used without prior purification. The organic solvents (ethanol, dichloromethane, hexane, and ethyl acetate HPLC obtained from Merck-Millipore) were used without prior purification.

The synthesis procedure reactions were conducted under the following conditions: A solution of o-phthaldialdehyde (3.0 mmol, 1.0 equiv), acetophenone (3.0 mmol, 1 equiv), and NaOH (7.0 mmol, 2.0 equiv) in 10 mL ethanol was stirred at 0 °C for 0.5 h under ultrasonic irradiation (UP200 St ultrasonic homogenizer, 200W, 26 kHz, Hielscher Ultrasonics).

#### 2.1.1 Purification Method A

After the reaction, 10 mL of water was added, and the pH was adjusted to two with an HCl solution (6M); it was then extracted with 15 mL of dichloromethane. The organic layer was dried over anhydrous Na_2_SO_4_, and the solvent was evaporated under a vacuum. The product was purified by flash column chromatography on silica gel 60 70–200 mesh using a mixture of hexane and ethyl acetate in a 98:2 to 97:3 (hexane/ethyl acetate) ratio as eluent. The yield of pure products is about 95–80%.

#### 2.1.2 Purification Method B

After the reaction, the pH was adjusted to two with an HCl solution (6M), and diluted hydrochloric acid was used to neutralize and precipitate the product. The precipitate was filtered out, washed with water, cold ethanol, and hexane, and dried as a pure product. The yield is about 85–70%. Using purification methodology B, the yield is slightly decreased. However, the product’s purity is higher. Due to the product’s low polarity, the column purification is extremely fast.

Compounds IND-1 (Z)-2-(hydroxy­(phenyl)­methylene)-2,3-dihydro-1H-inden-1-one, IND-2 (Z)-2-(hydroxy­(pyridine- 3-yl)­methylene)-2,3-dihydro-1H-inden-1-one and IND-3 (Z)-2-((4-(dimethylamino) phenyl)­(hydroxy) methylene)-2,3-dihydro-1H-inden-1-one have been reported previously, so their physical and spectroscopic data correlate with those in the literature.^42,48,49^ The molecular structures are shown in Scheme 2.

**Scheme 2:**
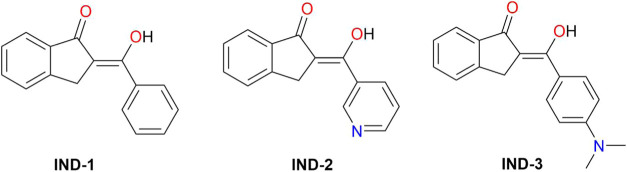
Molecular Structures of Indanone Derivatives Evaluated as Corrosion Inhibitors

The 2-benzylidene-1-indanone derivatives were characterized by nuclear magnetic resonance (NMR) of 1H and 13C acquired by Bruker D8 Venture k Geometry diffractometer 208039-1. The FT-IR by NICOLET IS-50, Thermo Fisher Scientific, obtained the FTIR spectra.

### 2.2 Evaluation of Indanone Derivatives as Corrosion Inhibitors

AISI 1018 samples were pickled with sandpaper, polished with cloth and alumina, cleaned with acetone, dried, and evaluated. Three different compounds dissolved in dimethyl sulfoxide (DMSO, 99% purity) acquired from Sigma-Aldrich were examined across five different concentrations (0, 0.03, 0.06, 0.12, and 0.25 M) to establish the most effective level of inhibition.

Electrochemical experiments were conducted on a 3-electrode cell: Ag/AgCl reference electrode, graphite rod as auxiliary electrode, AISI 1018 steel as working electrode with 1 cm^2^ contact area. A corrosive medium was prepared using 1 M hydrochloric acid from JP Baker (38% m/v purity). Gill AC potentiostat (ACM Instruments, UK) and Parallel software were used for data acquisition. First, an open circuit potential (OCP) measurement was conducted for 1800 s; thereafter, Electrochemical Impedance Spectroscopy (EIS) was applied at 10 mV AC amplitude at 10^4^ to 10^–2^ Hz with 10 points per decade. Finally, potentiodynamic polarization (PP) with an overpotential of ± 300 mV vs OCP with a sweep rate of 1 mV s^–1^. Assays were conducted in triplicate, and the standard deviation was reported to ensure reproducibility.

Tafel and EIS analyses were adjusted using EC-Lab V11.47 software. The inhibition efficiency (eq 1) was calculated from the corrosion current density (*i*_corr_) derived from the Tafel extrapolation, performed in the Tafel region (η = ±0.1 V) based on the ″best-fit value of *b*_a_ and *b*_c_, from the intersection of the lines.^50^

1 $$\eta =\left[{{(i}_{corr}}^{{}^{\circ}}-i_{c\mathrm{orr}}^{\mathrm{inh}})/{i_{corr}}^{{}^{\circ}}\right]\times 100$$

*i*_corr_^o^, corrosion density without an inhibitor, and *i*_corr_^inh^, corrosion density with an inhibitor.

The corrosion current density was calculated from the EIS results using the Stern-Geary equation, assuming activation control via the charge transfer resistance (*R*_ct_). The inhibition efficiency was determined using eq 2.

2 $$\eta =[\left(\frac{1}{R_{\mathrm{ct}}^{0}}\right)-\left(\frac{1}{R_{\mathrm{ct}}^{\mathrm{inh}}}\right)/\left(\frac{1}{R_{\mathrm{ct}}^{0}}\right)\times 100]$$

where *R*_ct_ blank indicates the absence of the inhibitor, and *R*_ct_ inhibitor suggests the presence of the inhibitor.

### 2.3 Adsorption Mechanism

Determining the adsorption isotherms of inhibitors on steel is essential for understanding metal-solution interactions. Langmuir isotherm (eq 3) was the fitting model.

3 $$\mathrm{Langmuir model}:\;C/\theta =1/K_{\mathrm{ads}}+C$$

*C*; concentration of the molecule, *K*_ads_; equilibrium constant of the adsorption process, ⊖; surface coverage. The surface coverage (⊖) values can be calculated using the following equation (eq 4).

4 $$\theta =1-(R_{\mathrm{ct}}^{{}^{\circ}}/R_{\mathrm{ct}}^{\mathrm{inhibitor}})$$

*R*_ct_^°^; charge transfer resistance in acid solution without inhibitor, *R*_ct_^inhibitor^ charge transfer resistance with inhibitor.

Langmuir adsorption isotherm fitting allowed accurate calculation of the Gibbs free energy according to eq 5.^51–55^

5 $$\Delta G=-\mathrm{RT}\;\ln\left(K\times C_{\mathrm{AP}}\right)$$

*R*, ideal gas constant (J/mol K); *T*, temperature (K); and *C*_AP_, concentration of pure water equals 55.55 mol/L, *K*, adsorption constant (L/mol).

### 2.4 SEM, EDS, and AFM Surface Characterization

Once the most effective concentration was determined, 4 samples were immersed in a 1 M hydrochloric acid solution for 1 week; one sample served as a control without inhibitor, and the other three had a concentration of 0.06 M. The coupons were then cleaned, dried, and inspected. Scanning electron microscopy (SEM) images and energy dispersive spectroscopy (EDS) analysis were performed using a JEOL JSM 5900-LV (JEOL, Japan) in a high vacuum at 15 kV for secondary electrons.

AFM analyses evaluated surface topography using NaioAFM equipment (Nanosurf, Switzerland) at a force of 18 nN and a scanning speed of 256 points per second within a 20 × 20 μm area. Samples were compared for the average roughness coefficient (*R*_a_), the mean line was calculated, and the rough profile was filtered from the raw profile data according to eq 6.

6 $$R_{a}=\frac{1}{n}\sum_{i=1}^{n}\left|Y_{i}\right|$$

### 2.5 FTIR Studies of Indanone Derivatives on Mild Steel

The IND adsorption was carried out using a mild steel surface immersed in a 1 M HCl solution with a 0.1 M compound for two hours, then cleaned and dried. IND-1, IND-2, and IND-3 FTIR spectra were recorded and compared with pure indanone derivatives. Subsequently, IND molecules were mixed with 1 M HCl solution, which were subjected to evaluation to acquire spectra of these, through diamond ATR-IR using Bruker α II Compact FT-IR Spectrometer.

### 2.6 Computational Analysis

Optimization calculations were done on the designed molecules using the hybrid functional B3PW91 implemented in the Gaussian 16 suite.^56^ In B3PW91, the exchange is incorporated through Becke’s parameter 3,^57^ while the functional Perdew and Wang provide the nonlocal correlation term. A 6–31G** basis set from the Gaussian 16 basis library was used in all calculations. Frequent calculations ensured that each optimized structure represented a local minimum on the potential energy surface. Some quantum chemical parameters including the highest occupied molecular orbital energy (*E*_HOMO_) and the lowest unoccupied molecular orbital energy (*E*_LUMO_), vertical ionization potential (*I*), the electron affinity (*A*), chemical potential (μ), electronegativity (ω), and hardness (η) were calculated.^58,59^ The eqs 7 to 10, for the parameters utilized based on the concept, are as follows

7 $$I={-E}_{\mathrm{HOMO}}$$

8 $$A={-E}_{\mathrm{LUMO}}$$

9 $$\mu =-\frac{I+A}{2}$$

10 $$\omega =\frac{\mu^{2}}{2\eta }$$

11 $$\eta =\frac{I-A}{2}$$

## 3 Results and Discussion

### 3.1 Synthesis and Characterization of Indanone Derivatives

The indanone derivatives (IND-1, IND-2, and IND-3) were synthesized following the procedure previously described by our research group with a slight modification. In this modification, an ultrasonic probe was employed as a nonconventional activation method, which allowed the compounds to be obtained in a shorter time and yield better than a conventional method.^60^ The compounds were characterized by comparing the HRMS data obtained with those described in the literature, results shown in Figures S2–S7 (Supporting Information).

Initially, the base structure (IND-1) was proposed as a study model, presenting a 2-benzylidene-1-indanone with a single aromatic ring, later contemplating the incorporation of functional groups that could modulate the electronic density along the system. For this purpose, the aromatic ring was replaced by a pyridine (IND-2), considering that this variation would modify the electron density in the 1,3-diketonate system, thereby reducing its coordination capacity with the metal. Finally, an electron donor group R = Me_2_N (IND-3) on the aromatic ring was added to obtain an electron-rich aromatic system. It is considered that the nitrogen of the dimethylamino substituent can introduce electron density into the π-conjugated system of the structural motif.

### 3.2 Assessing Corrosion Inhibitors

First, the open circuit potential (OCP) was monitored to reach a steady state before the electrochemical tests were performed. Figure S8 (Supporting Information) shows the variations in open circuit potential (OCP) of the AISI 1018 steel electrode in a 1.0 M HCl solution over time, with and without three different indanones at concentrations of 0.03, 0.06, 0.12, and 0.24 M. Steel stabilization occurred after 1000 s, with an average of −337.669 ± 12.081 mV vs Ag/AgCl. Analyzing the variation in the OCP response indicates that the compounds interact with the metal surface, likely due to adsorbed molecules. When IND-1 was added, the most stable potential at 0.06 M was −455.076 ± 1.505 mV vs Ag/AgCl, stabilizing after 600 s. IND-2 had a longer stabilization time, with maximum potential displacement also at 0.06 M, averaging −429.946 ± 20.905 mV vs Ag/AgCl after 1400 s. IND-3 stabilized after 1200 s, averaging −444.782 ± 11.604 mV vs Ag/AgCl at the same concentration. Figure 1 shows the PP of the three indanone derivatives evaluated as inhibitors.

**Figure 1:**
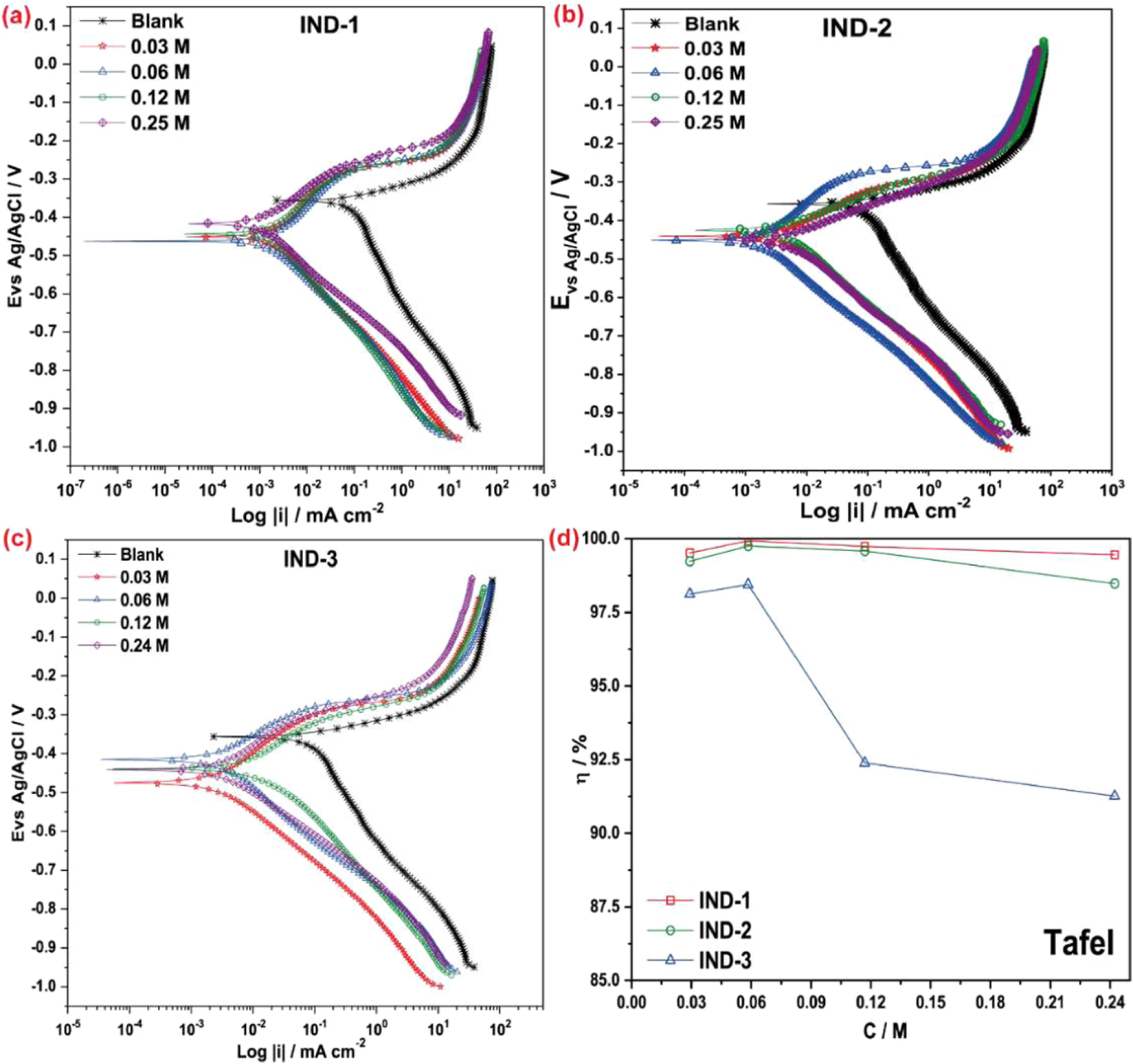
PP of AISI 1018 in 1.0 M HCl of (a) IND-1, (b) IND-2, (c) IND-3, and (d) Inhibitor efficiency.

Figure 1(a) for IND-1, 1­(b) for IND-2, and 1­(c) for IND-3 shows that adding the three molecules to the solution shifts the open circuit potential (OCP), reduces the cathodic reaction, and the corrosion current density (*i*_corr_) of carbon steel. Indanone acts as a cathodic inhibitor on metal surfaces by displacing the hydrogen reaction and blocking active sites at the metal–electrolyte interface, thereby reducing the cathodic reaction. Table S1 (Supporting Information) presents the data obtained from the polarization curve and the calculation of inhibitor efficiency compared to the mild steel without inhibitor.

The interaction of the compounds in the cathodic reaction significantly affects the corrosion kinetics, resulting in changes in slope compared to steel without inhibitor and reducing the corrosion current. At a concentration of 0.06 M, IND-1 has a cathodic slope of −261.4 ± 16.8 mV dec^–1^ and an *i*_corr_ of 0.227 ± 0.088 μA·cm^–2^. For IND-2, the slope is −248.6 ± 7.9 mV dec^–1^, with an *i*_corr_ of 0.967 ± 0.223 μA·cm^–2^. For IND-3, the slope is −267.2 ± 2.9 mV dec^–1^ and *i*_corr_ of 0.035 ± 0.009 μA·cm^–2^. Higher dosages do not improve the inhibition effectiveness of the three molecules, indicating that the electrochemical interaction relates to the metal–electrolyte interaction and lowers local pH, which reduces cathodic reactions.^61,62^

Electrochemical impedance spectroscopy (EIS) tests were also performed to compare the results obtained from potentiodynamic polarization and to discern the electrochemical contributions in the resistance and capacitance of each compound at the inhibitor-metal–electrolyte interface of AISI 1018 carbon steel. A significant increase in semicircles is noted in both the resistive (Zre) and capacitive (-Zim) components when the three indanones are added as inhibitors at any concentration. Unlike many organic compounds, this increase does not show a consistent progression related to concentration.

Figure 2 shows the Nyquist plots for AISI 1018 steel (a) and inhibitors IND-1 (b), IND-2 (c), and IND-3 (d).

**Figure 2:**
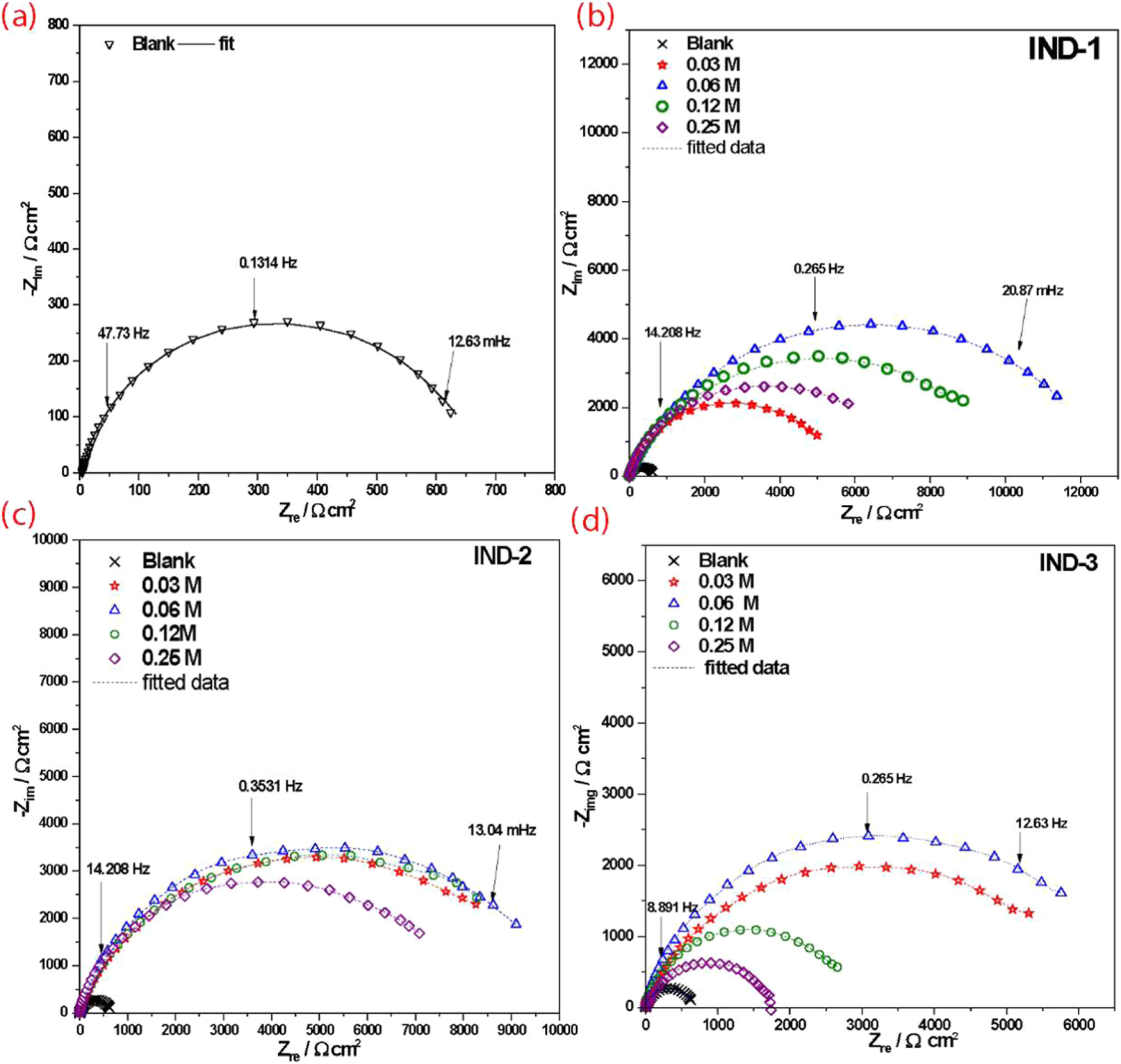
Nyquist plots for AISI 1018 in 1.0 M HCl of (a) Blank, (b)­IND-1, (c) IND-2, and (d) IND-3 at 298 K.

Figure 2(a) depicts the steel immersed in 1 M HCl, which reached a Zre value of 650 Ω·cm^2^, and a well-defined semicircle. This single time constant is related to the charge transfer resistance and electrochemical double-layer capacitance. In Figure 2(b), the plot shows the effect of different concentrations of the IND-1 compound. Figure 2(c), the IND-2 compound was also evaluated as a corrosion inhibitor. The same effect can be observed with a maximum Zre at 0.06 M concentration (9500 Ω·cm^2^). In Figure 2(d) for the IND-3 compound, the same Zre response is exhibited (6000 Ω·cm^2^). The increase in the value of Zre, which does not further increase as the inhibitor concentration increases, has practical implications for using indanones as corrosion inhibitors. Upon comparing the resistive components of the three indanones, the resistance decreases markedly in the presence of the pyridine substitution of the aromatic group in IND-2, and the dimethylamino group substituted in the chemical structure of IND-3 does not show an enhanced inhibition response compared to IND-1. Meanwhile, the dimethylamino group shows slightly lower resistance due to steric effects that hinder its ability to cover the metal surface.

Figure S9 (Supporting Information) shows the Bode modulus and Bode phase diagrams for the concentrations with the highest Zre in the Nyquist diagram (Figure 2). This detail enhances our investigation and helps us confidently select the best equivalent electrical circuit (EEC) for fitting. The comparative analysis of the impedance modulus increase was performed at low frequencies (10^–2^ Hz), assuming that charge transfer reactions are presented as the slowest controlling phenomenon. The blank sample presented an impedance modulus of 1,386.75 Ω·cm^2^. For IND-1, the impedance modulus was the highest with a value of 10,471.28 Ω·cm^2^, whereas for IND-2, it was 7943.28 Ω·cm^2^, and finally, for IND-3, it had a value of 3162.27 Ω·cm^2^.

Additionally, the blank demonstrates a phase shift of 55.57° at 101.7 Hz, with only one time constant noted; in this sense, the IND-1 sample shows a pronounced phase shift spread over the entire frequency range, reaching a maximum of 76.68° at frequencies above 300 Hz. This indicates the molecule’s action, revealing two simultaneous processes on the steel surface. A similar trend is observed for IND-2 and IND-3, indicating a capacitive, insulating response on the surface. This mechanism involves the selective adsorption of the compound in cathodic zones, increasing surface impedance and reducing species diffusion due to the molecules’ capacitive response. The EIS results were analyzed using equivalent electrical circuits (EEC) for quantitative assessment, as shown in Figure S10 (Supporting Information), corresponding to (a) steel without where *R*_sol_ is the solution resistance, *R*_ct_ is the charge transfer resistance, and *Q* is the constant phase element, and (b) steel with inhibitor suitable to describe the results in the presence of the inhibitor with the two associated time constants in series and other resistances that allow defining the adsorption parameters, such as *R*_mol_, which is the resistance of the organic molecules with its corresponding capacitance associated to the second time constant found in the corresponding Nyquist and Bode diagrams.^63^ *Q* was used to adjust the frequency phase shift of AC potential and current, as defined in the impedance representation in eq 12.^64^

12 $$C_{\mathrm{dl}}=Q^{1/\alpha }R^{\left(1-\alpha \right)/\alpha }$$

*C*_dl_ represents the double-layer capacitance, and ω is the angular frequency at which *Z*’ is at its maximum. Table S2 shows the electrochemical parameters of IND-1, IND-2, and IND-3 obtained by EEC fitting (Supporting Information).

The performed fitting for AISI 1018 steel without inhibitor is observed in which only a constant phase element was used with an α value of 0.877, indicating that the metal–electrolyte interface cannot be considered as an ideal capacitor and the amount of charge per unit area is determined with the frequency and resistance (*Q*_dl_) fit, giving an average value 64.55 ± 9.08 μF·cm^–2^ which suggested double layer values attributing a homogeneous corrosion phenomenon with a charge transfer resistance of 675 ± 29.45 Ω·cm^2^. The increase in *R*_ct_ (charge transfer resistance) with different concentrations of the inhibitors studied is attributed to a higher surface coverage by the organic molecules, leading to a higher inhibition efficiency. On the other hand, the decreased *Q*_dl_ (double-layer capacitance) of the column adsorption of organic molecules to the steel surface increases with concentration, thereby displacing water molecules at the metal-solution interface. A maximum increase of *R*_mol_ and *R*_ct_ and a decrease of *Q*_dl_, with a maximum value of, for IND-1 of 13 483 ± 70.145 Ω·cm^2^ was obtained, whereas for IND-2, an average value of 11 370 ± 147.342 Ω·cm^2^ and 9.97 ± 3.47 μF·cm^–2^ and finally for IND-3 a resistance value of 7 122 ± 190.047 Ω·cm^2^ and 20.37 ± 1.97 μF•cm^–2^. These values present the same concentration trend as described in the Tafel extrapolation, where the maximum efficiency was found at a concentration of 0.06 M for IND-1, 93.66% ± 0.51 for IND-2, and finally, 91.69% ± 0.99 for IND-3. To summarize, indanone molecules diminish current output by affecting the electrochemical double layer, which covers active sites on the metal surface at the metal–electrolyte interface, thereby hindering the movement of ions in the solution.

### 3.3 Adsorption Isotherm

EIS parameters were analyzed to understand the adsorption mechanism of indanone as an inhibitor. Generally, it is assumed that Δ*G*°_ads_ is related to the adsorption phenomenon. Physisorption (−20 kJ mol^–1^) involves electrostatic interactions between molecule charges and the electrode’s surface charge. In contrast, chemisorption (−40 kJ mol^–1^) involves charge transfer from inhibitor molecules to the metal surface through coordinated bonds.^65,66^ Figure S11 (Supporting Information) shows the Langmuir isotherm adjustment. The correlation coefficients and slope profile indicate that the experimental data are consistent, suggesting near-complete surface coverage and facilitating calculating the equilibrium constant and adsorption-free energy:^60,67^ Table 1 shows the data fitting indicates values related to the adsorption phenomenon.

**Table 1:** Adsorption According to the Langmuir Isotherm

molecule	interception 10^–7^	slope	correlation	*K*_ads_ 10^6^	Δ*G*_ads_ (kJ mol^–1^)	mechanism
IND-1	–4.387 ± 0.290	1.06619 ± 0.0025	0.9993 ± 0.0025	0.288	–45.616	chemisorption
IND-2	–6.048 ± 0.719	1.08234 ± 0.0023	0.9995 ± 0.0015	6.025	–42.426	chemisorption
IND-3	–68.956 ± 2.658	1.01677 ± 0.0021	0.9986 ± 0.0011	4.651	–39.396	physisorption-chemisorption

IND-1’s high energy levels result from the absence of heteroatoms in its aromatic rings, allowing for uniform electron delocalization. Iron’s empty d orbitals interact with the diffuse electron cloud, leading to stronger π-d bonds and more effective adsorption onto the metal surface.^68^ The structural motif of 1,3-diketonate in all three molecules is essential for chemisorption’s significant effect.

The presence of the N heteroatom in IND-2 and IND-3 does not favor π-π stacking between adjacent molecules of the inhibitor because the homogeneity in the electronic distribution is modified, as well as the planarity of the molecule, this effect being of more significant impact in IND-3, given the presence of the dimethylamino group. Such stacking forms a more organized, dense, and stable protective film on the metal surface when only the aromatic ring in IND-1 is present, acting as a more effective barrier against the penetration of corrosive substances, thus preventing steel corrosion.^69^ When the pyridine ring is present in IND-2, the primary interaction with the steel is based on the formation of π-d bonds between the π electronic cloud of the ring and the empty *d* orbitals of the iron, which allows the adsorption of the pyridine ring on the steel surface, forming a protective layer that inhibits corrosion.^70^

The dimethylamino group in IND-3 reduces interactions between the aromatic ring and the 1,3-diketonate system, lowering the inhibition efficacy. The rotation of the dimethylamino group inhibits molecular stacking and effective absorption on the π-d metal orbitals. A kinetic barrier is established, leading to a decrease in surface interaction and an increase in intrinsic binding energy, which inhibits molecule adsorption.^71–73^

### 3.4 Study of Surface Morphology

After determining the optimal concentration for each indanone, immersion tests were performed on carbon steel for 1 week to assess the fouling from corrosion products and changes in morphology. Figure 3 shows the corrosion behavior of steel without an inhibitor (blank) and with 0.06 M concentrations of each indanone. These results highlight the efficacy of the indanones in inhibiting carbon steel corrosion under real-world conditions.

**Figure 3:**
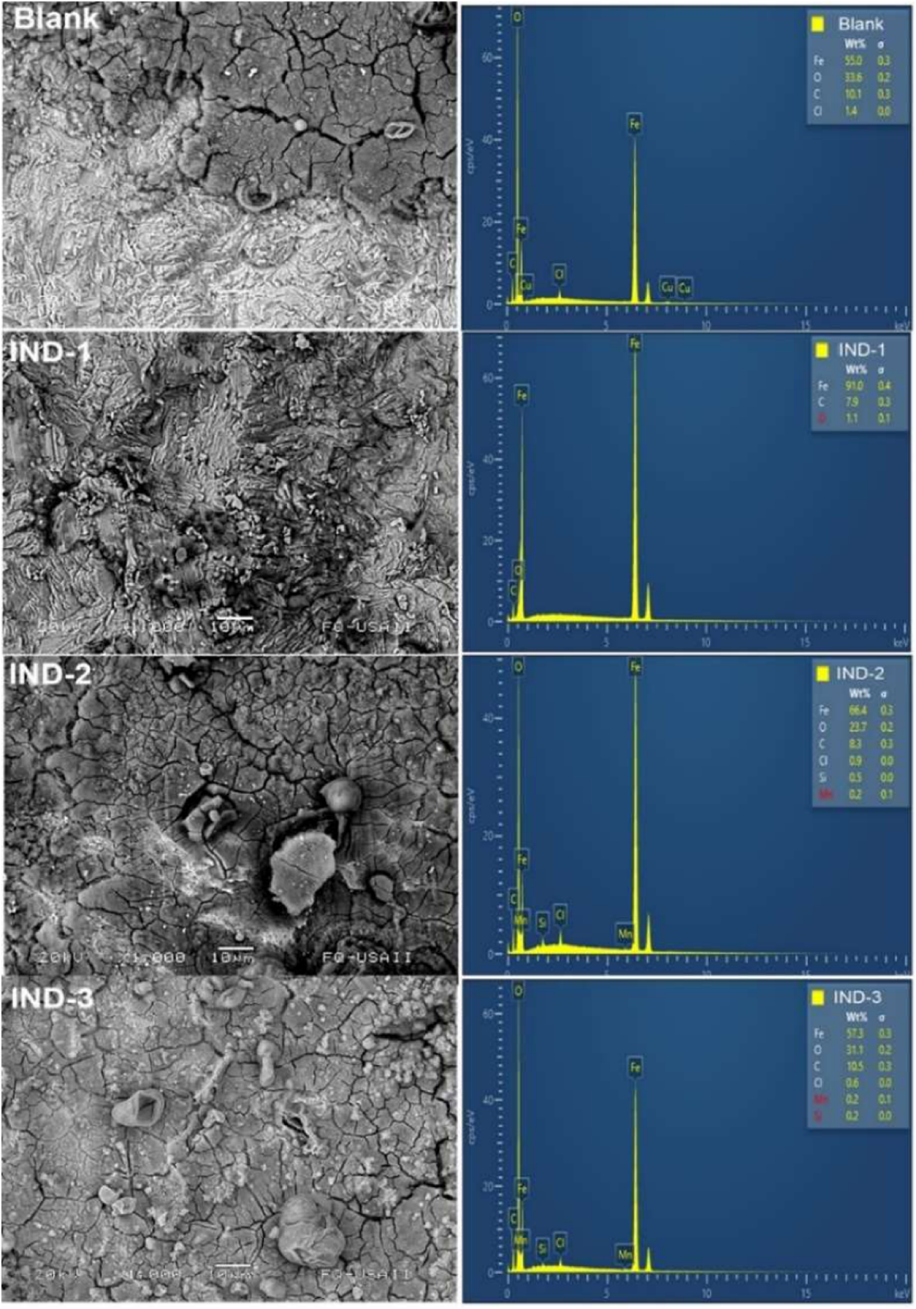
AISI 1018 steel without inhibitors and IND-1, IND-2, and IND-3 at 0.06 M.

The steel coupons clearly distinguish between a corrosion-free area and a corroded section. Corrosion occurs unevenly at the grain edges, indicating localized corrosion and possibly pitting. The circular shapes and deeper holes suggest embedded crystalline chloride products, with active sites exposed to the environment, leading to chloride salts and oxygen buildup. The mechanism is controlled by species diffusion at the oxide-metal interface, with microanalysis showing oxygen (33.6%) and chlorine (1.4%) concentrations. A comparison of the morphology and corrosion products for IND-1 shows that AISI 1018 steel has a uniform surface depth with no localized zones or distinct grain boundaries. Corrosion-free areas demonstrate the effective action of IND-1 indanone, showing an oxygen level of 1.1% and no chloride detected.

Furthermore, IND-2 has a darker area due to surface variations, and the lighter regions suggest minimal corrosion caused by chloride (Cl 0.9%) and oxygen (O 23.7%), which are lower than without inhibitors. In contrast, IND-3 shows delamination and localized corrosion, with 31.1% oxygen and 0.3% chlorine detected. The evaluated indanones showed a strong inhibitory effect by creating a protective layer against steel corrosion, supporting the electrochemical findings. While this compound mitigates hydrochloric acid effects and chloride ion attacks, it is less effective at inhibiting oxygen diffusion to the surface than the other two compounds.

The research goal relies on the surface roughness information, with the average roughness (*R*_a_) calculated using AFM data as the comparison parameter. The arithmetic means of the absolute values of the roughness profile ordinates is commonly adopted in general engineering practice.^74^ The height is considered positive in the upward direction, moving away from the bulk material from the bulk material. Indicates the AFM images of the corroded and inhibited steel alloy surface. Some of them are *R*_a_, the average surface roughness, shown by the arithmetic mean height; *R*_q_, the root mean square roughness; and *R*_max_, the average of the peak separation profile’s irregularities, as shown in Figure 4. AFM was used to obtain more detailed information on the influence of indanones on the level of destruction or damage to the steel surface at surface leveling. Table S3 (see Supporting Information) shows the average roughness (*R*_a_), root-mean-square (*R*_q_) values, and observed roughness of the steel alloy evaluated at 0.06 M of IND-1, IND-2, and IND-3.

**Figure 4:**
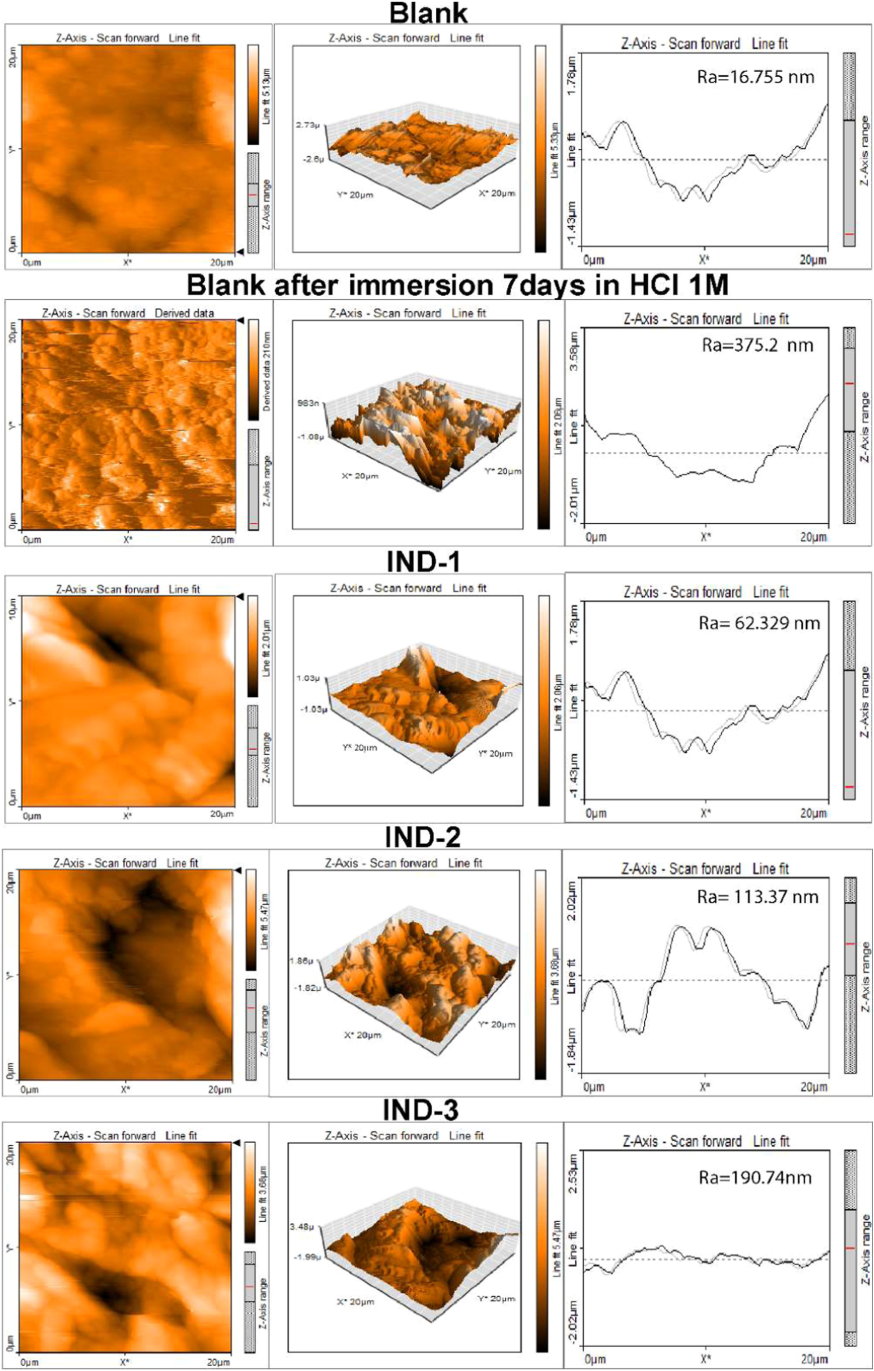
AFM of AISI 1018 mild steel without inhibitor, IND-1, IND-2, and IND-3 at 0.06 M.

The polished AISI 1018 steel’s average surface roughness before immersion was 18.755 nm. After immersion, in the absence of an inhibitor (Blank), the steel presents more significant depth irregularities and specific surface patterns with prolonged irregularities due to the uncontrolled corroded surface, which has a mean roughness of 375.2 nm.

As per the presented data, all samples treated with indanones exhibit a reduced *R*_a_ parameter compared to the roughness coefficient of the 1018 steel samples. The lowest roughness coefficients were observed for samples with IND-1, with a roughness coefficient of 62.324 nm. For IND-2, there is an increase in *R*_a_ of 113.37 nm, and finally, for IND-3, a *R*_a_ of 190.74 nm. The mean roughness (*R*_a_), root-mean-square (*R*_q_) values, and observed roughness of the steel alloy immersed in 0.06 M of IND-1, IND2, and IND-3 are much lower than those of the sample immersed in 0.1 M HCl without inhibitor. AFM analysis showed that exposing steel to an acidic medium resulted in a rougher surface, indicating increased damage. In contrast, the presence of indanones smoothened the surface, suggesting they formed a protective film by reducing roughness and texture.

### 3.5 FT-IR Characterization of IND-1, IND-2, and IND-3

FTIR spectra of pure IND-1, IND-2, and IND-3 are shown in Figures S12–S14 (see Supporting Information). As illustrated in Figure S14, the infrared spectrum for the pure IND-1 can be seen. At 3060 cm^–1^, the band indicates asymmetric and symmetric stretching of the various C–H groups. The adsorption band at 1603 cm^–1^ characterizes the C=O group of β-diketone in its enol form with an intramolecular H-bond. Similar bands are shown in IND-2 and IND-3 with differences due to the presence of C=N (pyridine core) and C-R_2_ (dimethylamino group) bonds, respectively. Figure S15 examines the film designed on a mild steel surface. Substantial changes can be observed between the C=O group shifting from 1603 cm^–1^ to 1657 cm^–1^ and the C–H group shifting from 3060 cm^–1^ to 3002 cm^–1^. The fact that some bands of indanone derivatives are absent suggests the adsorption of the inhibitor on Fe. Comparable band shifts are observed in IND-2 and IND-3. Figure S16 shows the film designed on a steel surface, the infrared spectrum of IND-3. 3524 cm^–1^, a broad band indicates the NR_2_ group presence and at 3439 cm^–1^, a broad band indicates the OH group. Figures S15–S17 show the interaction of IND-1, IND-2, and IND-3 with a 1 M HCl solution; for IND-3, changes were observed between the NR_2_ group, moving from 3524 to 3705 cm^–1^, suggesting protonation of the NR_2_ group in the HCl solution.

### 3.6 Computational Theory

A quantum analysis was performed to better understand the corrosion inhibition behavior from experimental data. The indanones and their electronic distribution allow for the description of how functional groups tend to transfer electrons, and quantum parameters help deduce the reactivity of these species. The boundary molecular orbital scheme for IND-1, IND-2 and IND-3 of the electron density distributions in the highest occupied molecular orbital (HOMO) and lowest unoccupied molecular orbital (LUMO) are shown in Figure 5.

**Figure 5:**
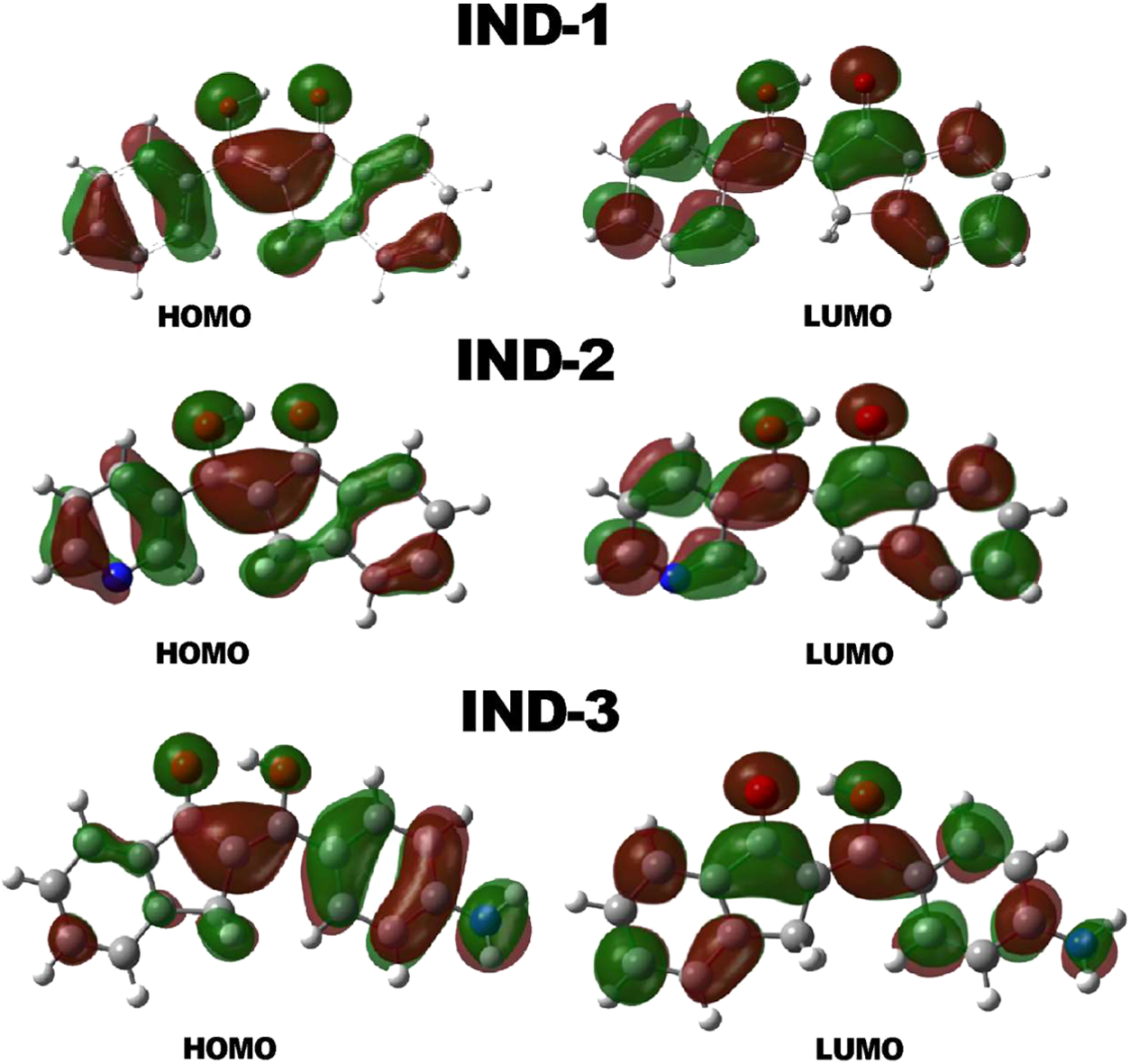
Frontier molecular orbitals of IND-1, IND-2, and IND-3 compounds.

Electron-donating (ED) and electron-withdrawing (EW) substituents affect electron density in molecules, influencing their adsorption and orientation on metal surfaces; substituents enhance the HOMO and/or LUMO contributions, while electron donors reduce them.

The HOMO lobes are located mainly from the C–H groups between the oxygen atom of the carbonyl functional and the hydrogen atom corresponding to the hydroxyl group. The LUMO functions remain practically unaffected after the substitutions concerning the shape. However, the position on the MO diagram presents significant differences, which are discussed below. ED substituents boost orbital contributions (HOMO and LUMO) for enhanced metal interaction, while EW ones reduce these interactions, impacting inhibition potential. ED promotes a planar orientation, whereas EW causes a vertical orientation.^40^ The LUMO lobes comprise a large center that the pyridine and dimethylamino functional groups have modified. The interaction was studied using the Wiberg index and Grimme modules, with an average bond length of 1.59 Å, suggesting a significant interaction. The Grimme modulus gives an average 18.5 kcal/mol value for this noncovalent interaction. Also, the average Wiberg index is 0.133, which is considerable compared to other studies.^75–77^

Parameters derived from the orbitals were calculated from the negative values of HOMO and LUMO; results correspond to the IND-1. IND-2 and IND-3 are shown in Table S4 (Supporting Information). Electronegativity is the most critical parameter to consider, as a significantly high value of this property implies a lower ability to cover the steel surface, and it can be taken as an electrophilicity index.^58,78,79^ The calculation suggests that the molecules do not have a uniform electron density distribution around the molecule, indicating that the energy asymmetry comes from the aggregated functional groups, confirming the reason for the decrease in the adsorption mechanism.^80–82^ Consequently, it demonstrates that organic anticorrosion agents can form covalent complexes with the iron network, with IND-1 being the best choice as an anticorrosion agent, providing an electron-rich zone to protect the iron surface.

It is essential to study the interaction of these compounds with an iron object to evaluate their anticorrosive properties. An iron complex was chosen for testing, representing a cubic cell with a regular cubic lattice pattern centered on faces (fcc) (Figure. [Fig fig6](a)); this model and a complex formed by this sample and one of the organic agents molecular electrostatic potentials assigned to the electron density of indanone compounds are shown in Figure 6(b) to IND-1, Figure 6(c) to IND-2 and Figure 6(d) for IND-3.

**Figure 6:**
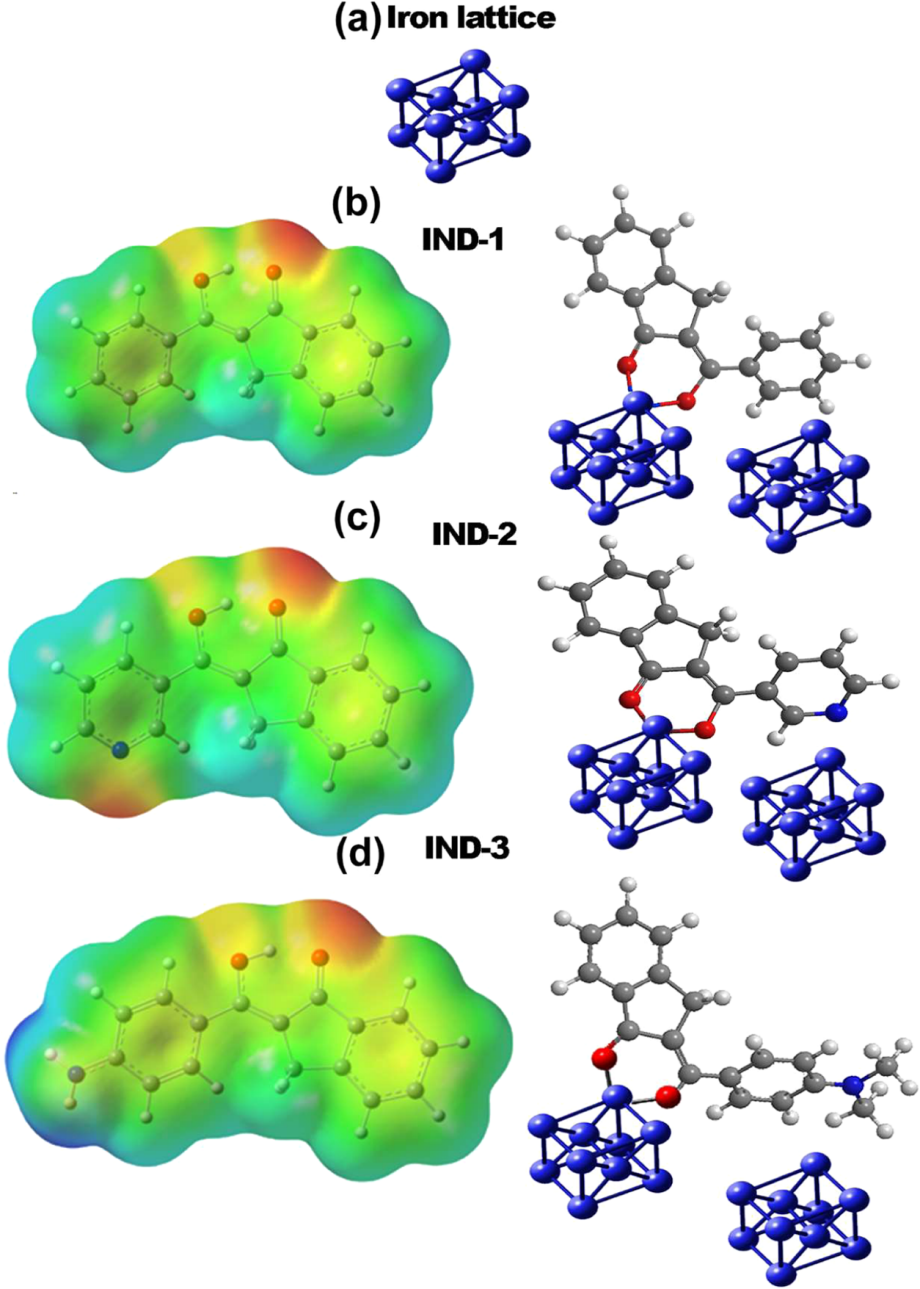
Molecular electrostatic potential mapped onto the electron density and simultaneous interaction of carboxylic group and aromatic ring with Fe particles of (a) face-centered cubic iron lattice, (b) IND-1, (c) IND-2, and (d) IND-3.

Again, this is the crucial involvement of the pyridine ring due to its lone elements. The molecular electrostatic potential assigned to the electron density of compounds is also visible and clearly shows a red (electron-rich) region that may offer this disadvantage for molecule–surface interactions. The prominent feature is the negative solid density near the lone electron pair of pyridinic nitrogen atoms and the border molecular orbitals of compound IND-2.

Jiménez-Cruz et al.^46^ suggested that the lateral aromatic ring interacts with the bond, forming a π-interaction with the Fe surface, simulating the interaction of the carboxylic group and the aromatic ring with the atoms on the Fe surface, positing that the ring could interact with the bond and the carboxylic group simultaneously. In contrast to their work, our research focuses on interactions with independent Fe cubes, where a stable complex with geometry like a ferrocene platform was identified. The sum energy of the six bonds and the average Wiberg index are shown in Table 2, where the bond length (bl), the Wiberg index (WI) for the Fe–O bond, and the energy value (E) of the specific bond itself are shown.

**Table 2:** Bond Length (bl), Wiberg Index (WI), and Coordination Covalent Bond Energy (E) of the Anticorrosion Agents

compound	Bl (Å)	WI	*E* (kcal mol^–1^)
IND-1	1.93	0.89	7.6
IND-2	1.98	0.81	7.1
IND-3	2.02	0.78	6.8
Aro-Fe	1.89	0.23	5.7

The results highlight that compounds IND-1 and IND-2 present the same bond distance between each carbon atom of the aromatic ring with the atypical Fe atom in the cube, and IND-1 is best suited to achieve a well-coordinated covalent bond and agree with the calculated electronegativity.^83^ In contrast, the bond length between IND-3 and the metal surface is the longest, suggesting that this distance enhances the attraction of the molecule to the surface, reinforcing the existence of formed compounds. The aromatic configuration forces the indanones to have a planar conformation, facilitated by the hydrogen bonding interaction between the carbonyl’s oxygen and the hydroxyl group’s hydrogen.^84,85^ In summary, IND-3 is the definitive attractor, while IND-1 is the leading oxidation inhibitor.

### 3.7 Inhibition Mechanism of 2-Benzylidene-1-Indanone Molecules

Based on 1H-NMR experimental and theoretical analysis, tautomeric equilibrium allows the enol-keto tautomer to be found in a ratio greater than 90% (see Supporting Information S3, S5, and S7). The adsorption mechanism of the molecules on the steel surface is proposed, considering their interaction, as shown in Figure 7.

**Figure 7:**
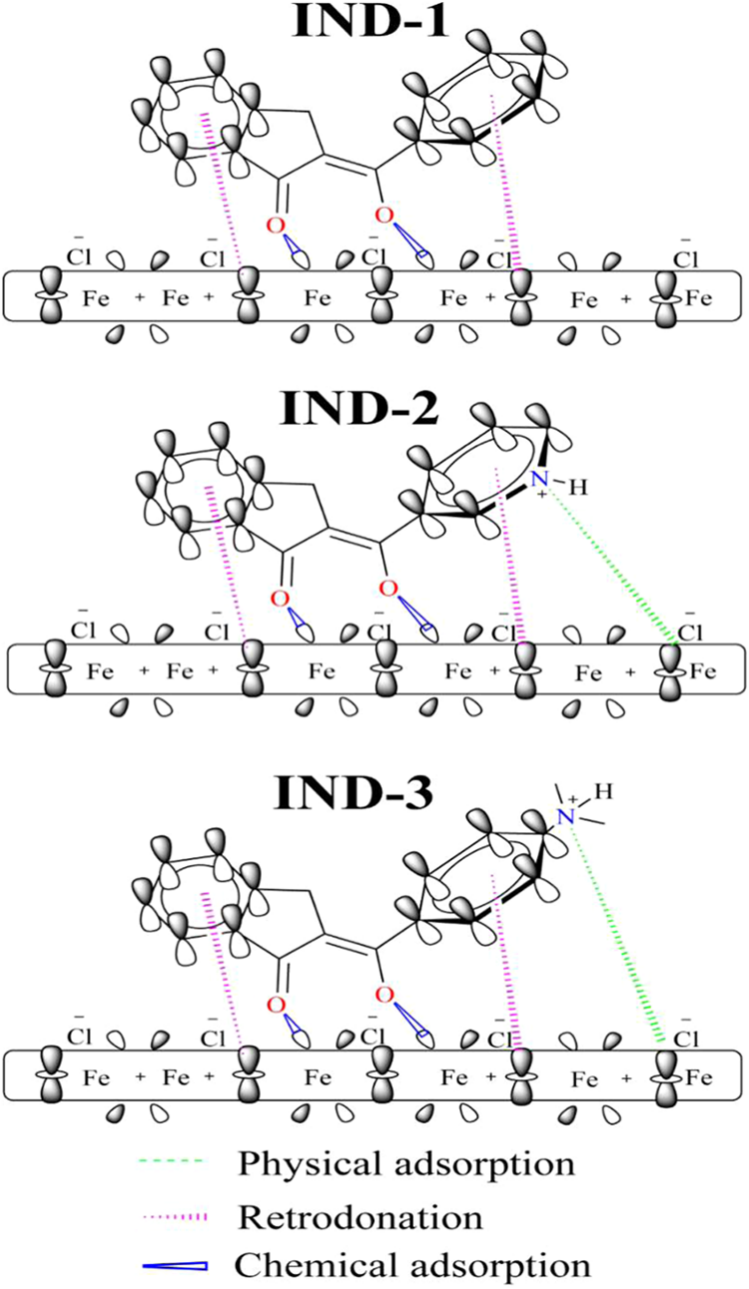
Absorption mechanism of IND-1, IND-2, and IND-3 in mild steel.

It has been reported that organic molecules containing heteroatoms in their molecular structure are readily protonated when immersed in 1.0 mol L^–1^ HCl;^86,87^ photographic evidence and mechanism of pyridine ring protonation are shown in Figure S18 (Supporting Information). The thermodynamic properties of adsorption and quantum calculations indicated the existence of chemical adsorption phenomena and charge transfer of free electron pairs on the hydroxyl and carbonyl group atoms via donor–acceptor interactions between the lone electron pairs of heteroatoms and the π electrons of multiple bonds with the vacant d orbitals of steel, supported by IR shown in Figures S13–S18 (Supporting Information).^88–92^ Consequently, the adsorbate molecules assume a planar conformation that secures the metal substrate. In the case of IND-1, which exhibits the best efficiency and lowest chemical adsorption energy, the inhibition mechanism may be attributed to the fact that the group functions are capable of undergoing protonation in hydrochloric acid (CH_3_NH_2_H^+^), thereby acquiring a positive charge, which facilitates the formation of electrostatic interactions with the steel surface, typically characterized by a negative charge in corrosive media.^93–95^ A reduction in the effectiveness of the IND-2 and IND-3 mechanisms may be attributed to the attraction of interactions with the surrounding solution or to the positive charge acquired by the steel surface following the loss of electrons. These molecules obstruct the transfer of electrons from the steel surface to the antibonding orbitals, a process known as retrodonation, and the retrodonation rate is determined by the number of functional groups or aromatic rings present at.^96–100^

## 4 Conclusions

This study successfully synthesized three 2-benzylidene-1-indanone derivatives using ultrasonic conditions. This method efficiently decreases reaction times and energy usage while minimizing waste and promoting environmental sustainability.

Based on the electrochemical techniques used to assess inhibitors, it was discovered that IND-1 > IND-2 > IND-3 were the orders of effectiveness and designated as a cathodic inhibitor. The hydroxy indanones formed a protective film on the surface of AISI 1018 steel, which prevented the metal from dissolving in the corrosive medium. The Langmuir model indicates that the interaction with the metal surface involves chemisorption for IND-1 and IND-2, while IND-3 exhibits both physisorption and chemisorption.

Modifying the aromaticity of 2-benzylidene-1-indanone structures increases the electronegativity and electron density, even though they reduce their adsorption capacity on the π-conjugated iron orbital. The theoretical results and experimental findings coincide, indicating that compound IND-1 is the most potent inhibitor, and compound IND-3 may be a better choice for an attractant.

## Supplementary Material



## Notes

Schemes of synthesis of 2-benzylidene-1-indanone derivatives; spectra HRMS of IND-1, IND-2 and IND3 in CDCl3 300 MHz; OCP vs t plot; potentiodynamic polarization parameters for corrosion of mild steel in 1 M HCl; Bode Modulus and Bode Phase plots at 0.06 M indanones; equivalent circuit model; electrochemical parameters fit for indanone derivatives; Langmuir Isotherm plot; AFM data of carbon steel without inhibitor, IND-1, IND-2, and IND-3; comparison FTIR spectrum of pure indanones derivatives and scrapped sample from the mild steel surface IND-1, IND-2 and IND-3; values of electronegativity of theoretical (eV) anticorrosion agents; photogram and mechanism of pyridine ring protonation (PDF)
