# Supplementary material for: Ultrasound-Assisted Synthesis of 2‑Benzylidene-1-Indanone Derivatives and Evaluation as a Corrosion Inhibitor for Mild Steel in 1 M HCl Solution
Source: ACS Omega. 2025 May 20;10(21):21147–61. doi: 10.1021/acsomega.4c09705 (PMC12138709; doi:10.1021/acsomega.4c09705)
Supplement: Supplementary file 1 [file ao4c09705_si_001.pdf]

Ultrasound-assisted synthesis of 2-benzylidene-1-indanone derivates and evaluation as a corrosion inhibitor for mild steel in 1 M HCl solution

Ricardo Ballinas-Indili [a], *Paola Roncagliolo-Barrera* [b], *Roberto Salcedo* [c], *Francisco J. Rodríguez-Gómez* [b], *Cecilio Álvarez-Toledano* [d]

[a] Departamento de Ciencias Químicas, Facultad de Estudios Superiores Cuautitlán Campo 1, Universidad Nacional Autónoma de México, Estado de México, 54740, México.

[b] Departamento de Ingeniería Metalúrgica, Facultad de Química, Ciudad Universitaria, Ciudad de México, 04510 , México.

[c] Instituto de Investigaciones en Materiales, Universidad Nacional Autónoma de México, Ciudad Universitaria, Ciudad de México, 04510, México.

[d] Instituto de Química, Universidad Nacional Autónoma de México, Ciudad Universitaria, Ciudad de México, 04510, México.

Corresponding author:* Tel.: +52 5540068294. Email address: proncagliolo@quimica.com.mx

SUPPORTING INFORMATION

Content of Material Provided as Supporting Information

1. List of Figures

1.1. Figure S1. Synthesis of 2-benzylidene-1-indanone derivatives.
1.2. Figure S2. HRMS of IND-1.
1.3. Figure S3. ^{1}H-NMR spectrum of IND-1 in $CDCl_3$ 300 MHz.
1.4. Figure S4. HRMS of IND-2.
1.5. Figure S5. ^{1}H-NMR spectrum of IND-2 in CDCl3 300 MHz.
1.6. Figure S6. HRMS of IND-3.
1.7. Figure S7. ^{1}H-NMR spectrum of IND-3 in CDCl3 300 MHz.
1.8. Figure S8. OCP vs. t of AISI 1018 in 1.0 M HCl of IND-1, IND-2, IND-3 at 298 K.
1.9. Figure S9. Bode Modulus and Bode Phase plots of the Blank (black line), IND-1 (red line), IND-2 (green line) and IND-3 (blue line) at 0.06 M.
1.10. Figure S10. Equivalent electrical circuits were used to fit the experimental data.
1.11. Figure S11. Langmuir Isotherm for the indanone compounds at 298 K.
1.12. Figure S12. FTIR spectrum of Fe (mild steel), pure IND-1 and steel of IND-1.
1.13. Figure S13. FTIR spectrum of Fe (mild steel), pure IND-2 and steel of IND-2.
1.14. Figure S14. FTIR spectrum of Fe (mild steel), pure IND-3 and steel of IND-3.
1.15. Figure S15. FTIR spectrum of IND-1 in 1M HCl solution.
1.16. Figure S16. FTIR spectrum of IND-2 in 1M HCl solution.
1.17. Figure S17. FTIR spectrum of IND-3 in 1M HCl solution.
1.18. Figure S18. Photogram and Mechanism of pyridine ring protonation of a) 1M HCl, b) IND-1 in 1M HCl, c) IND-2 in 1M HCl, and IND-3 in 1M HCl.

2. List of Tables

2.1 Table S1. Tafel extrapolation at IND-1, IND-2, and IND-3.
2.2 Table S2. Electrochemical parameters fit EEC for indanone derivatives.
2.3 Table S3. AFM data of carbon steel without inhibitor, IND-1, IND-2, and IND-3.
2.4 Table S4. Electronegativity of theoretical (eV) anticorrosion agents.

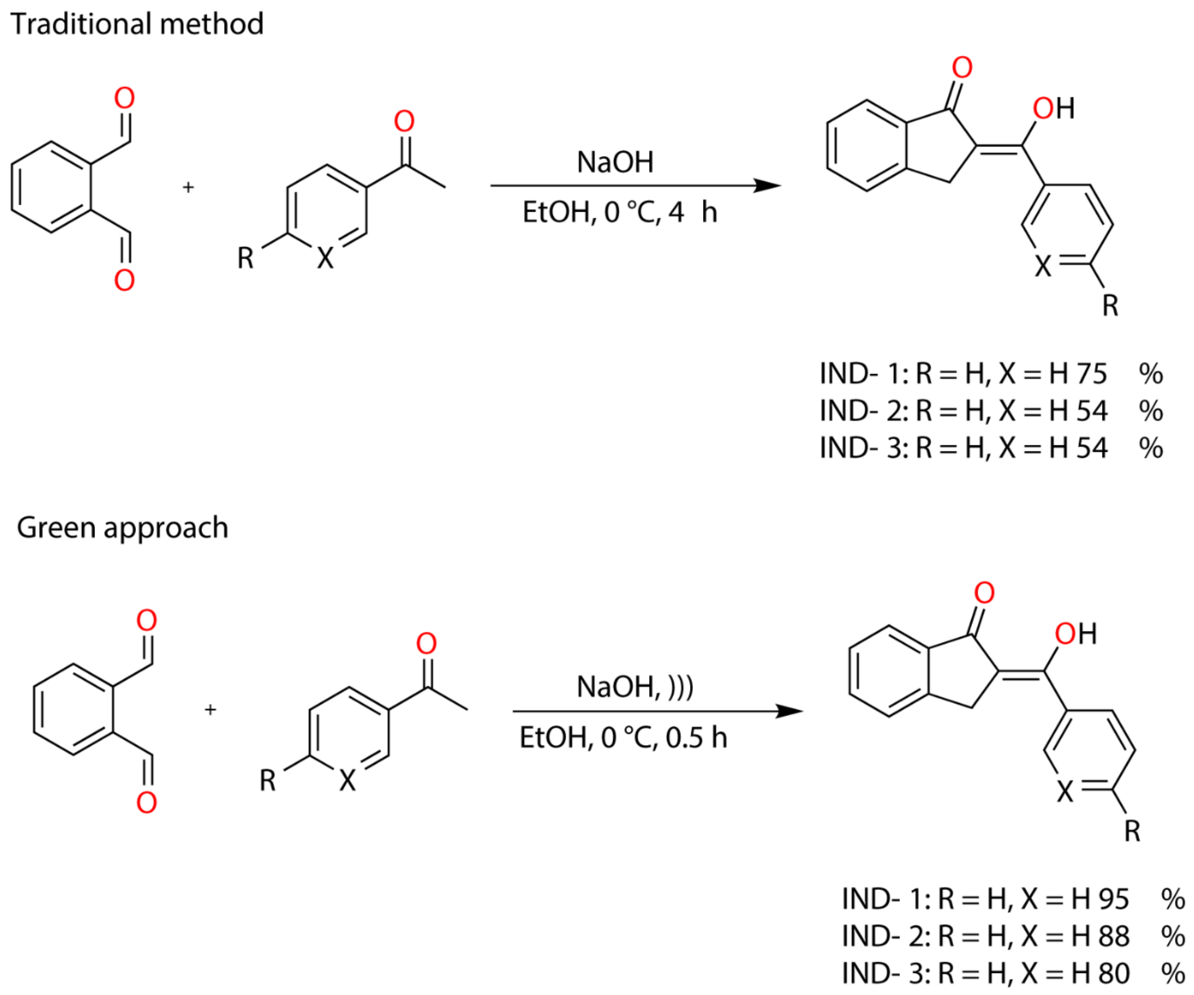

Figure S1. Synthesis of 2-benzylidene-1-indanone derivatives.

Description:
Ionization Mode:ESI+
History:Determine m/z[Peak Detect[Centroid,30,Area];Correct Base[];Smooth[5]];Correct Base[5.0%];Average(MS[...

Mass Calibration data:JeolCalibration
Created:1/12/2024 12:37:21 PM
Created by:AccuTOF

Charge number:1
Element:^{12}C:0 .. 18, ^{1}H:0 .. 20, ^{14}N:0 .. 0, ^{16}O:0 .. 3

Tolerance:5.00(ppm), 5.00 .. 15.00(mmu)

Unsaturation Number:-1.0 .. 40.0 (Fraction:Both)

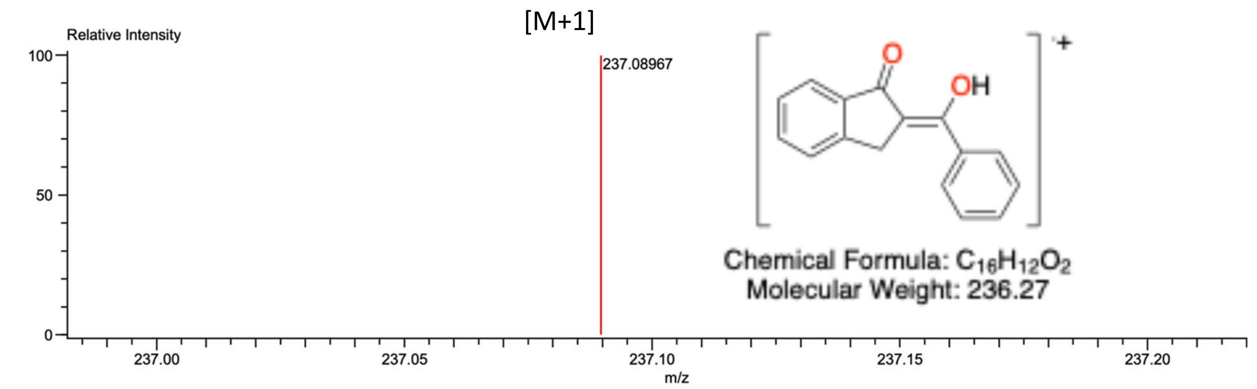

Mass	Intensity	Calc. Mass	Mass Difference (mmu)	Mass Difference (ppm)	Possible Formula	Unsaturation Number
237.08967	150178.22	237.09155	-1.88	-7.95	$^{12}C_{16}{}^{1}H_{13}{}^{16}O_2$	10.5

Figure S2. HRMS of IND-1 (DART 18.5 eV).

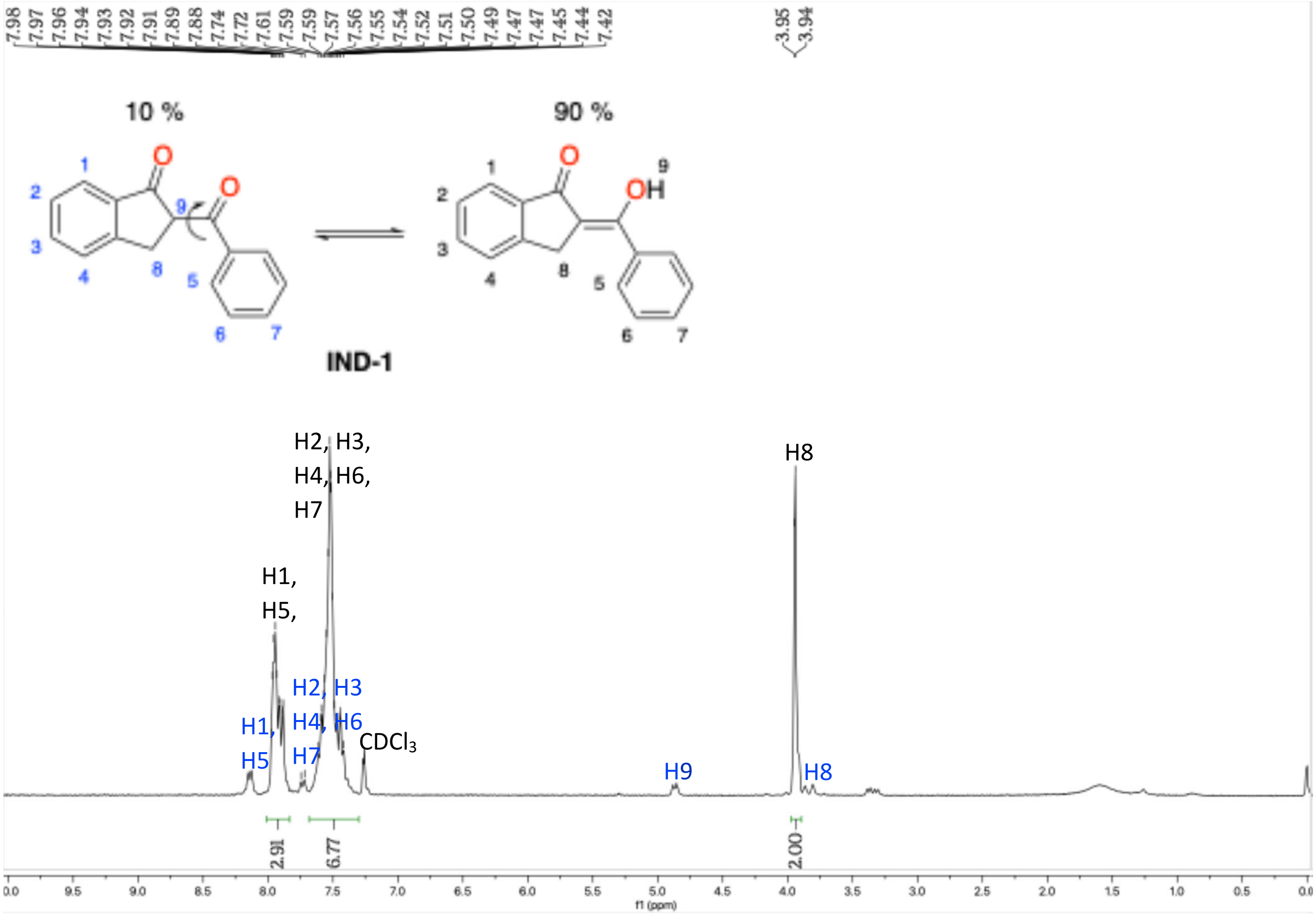

Figure S3. ^{1}H-NMR spectrum of IND-1 in $CDCl_3$ 300 MHz.

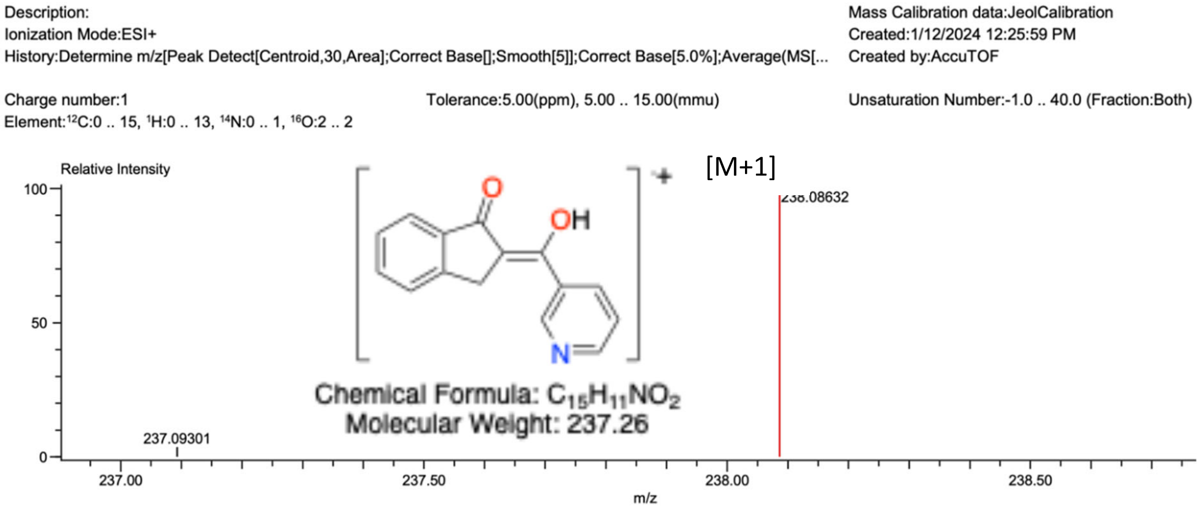

Mass	Intensity	Calc. Mass	Mass Difference (mmu)	Mass Difference (ppm)	Possible Formula	Unsaturation Number
238.08632	124120.30	238.08680	-0.48	-2.01	$^{12}C_{15}{}^{1}H_{12}{}^{14}N_{1}{}^{16}O_{2}$	10.5

Figure S4. HRMS of IND-2 (DART 18.5 eV).

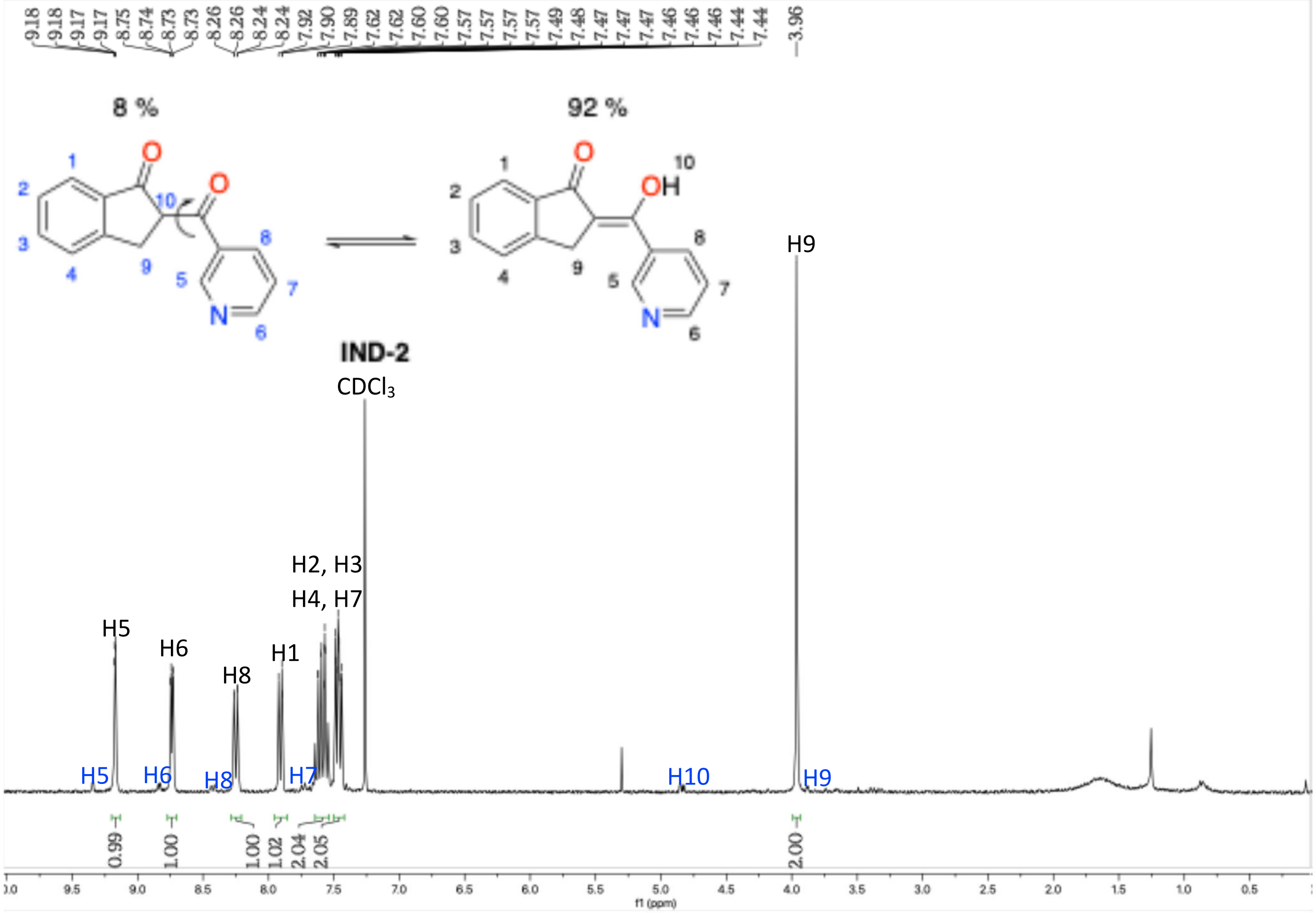

Figure S5. ^{1}H-NMR spectrum of IND-2 in $CDCl_3$ 300 MHz.

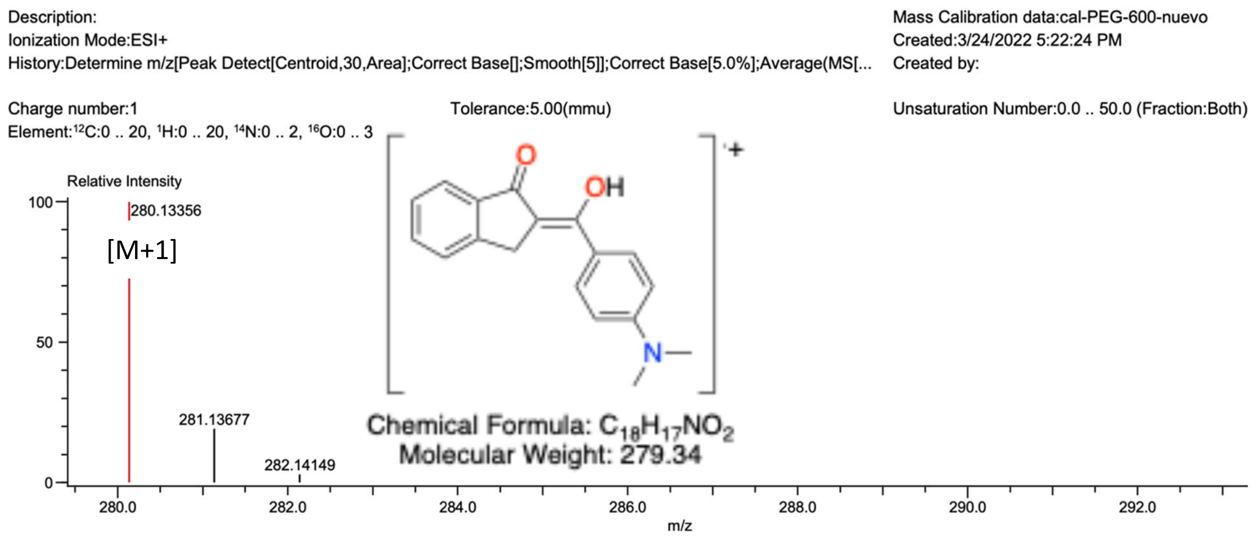

Mass	Intensity	Calc. Mass	Mass Difference (mmu)	Mass Difference (ppm)	Possible Formula	Unsaturation Number
280.13356	636194.82	280.13375	-0.20	-0.70	$^{12}C_{18}{}^{1}H_{18}{}^{14}N_{1}{}^{16}O_{2}$	10.5

Figure S6. HRMS of IND-3 (DART 18.5 eV).

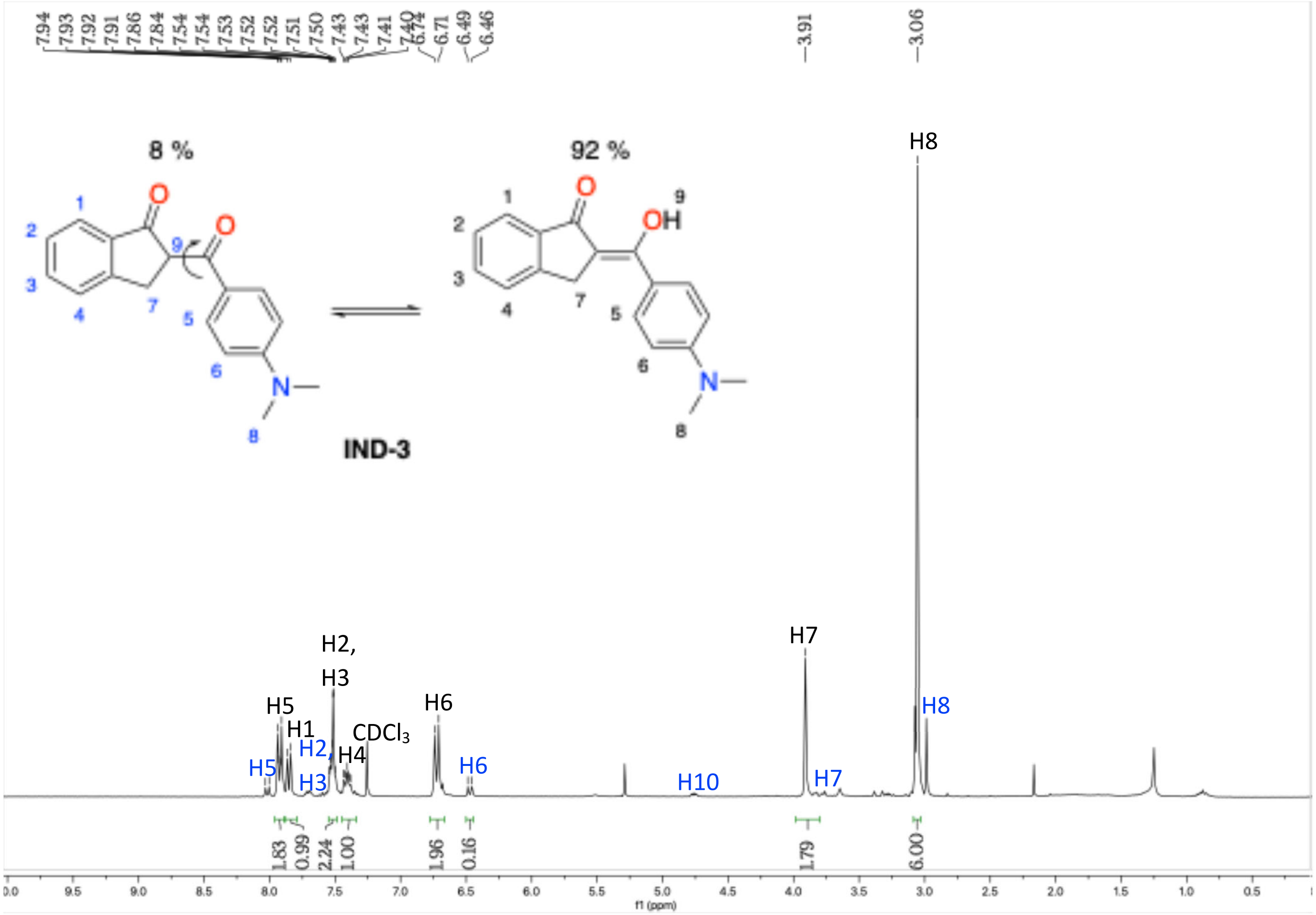

Figure S7. ^{1}H-NMR spectrum of IND-1 in $CDCl_3$ 300 MHz.

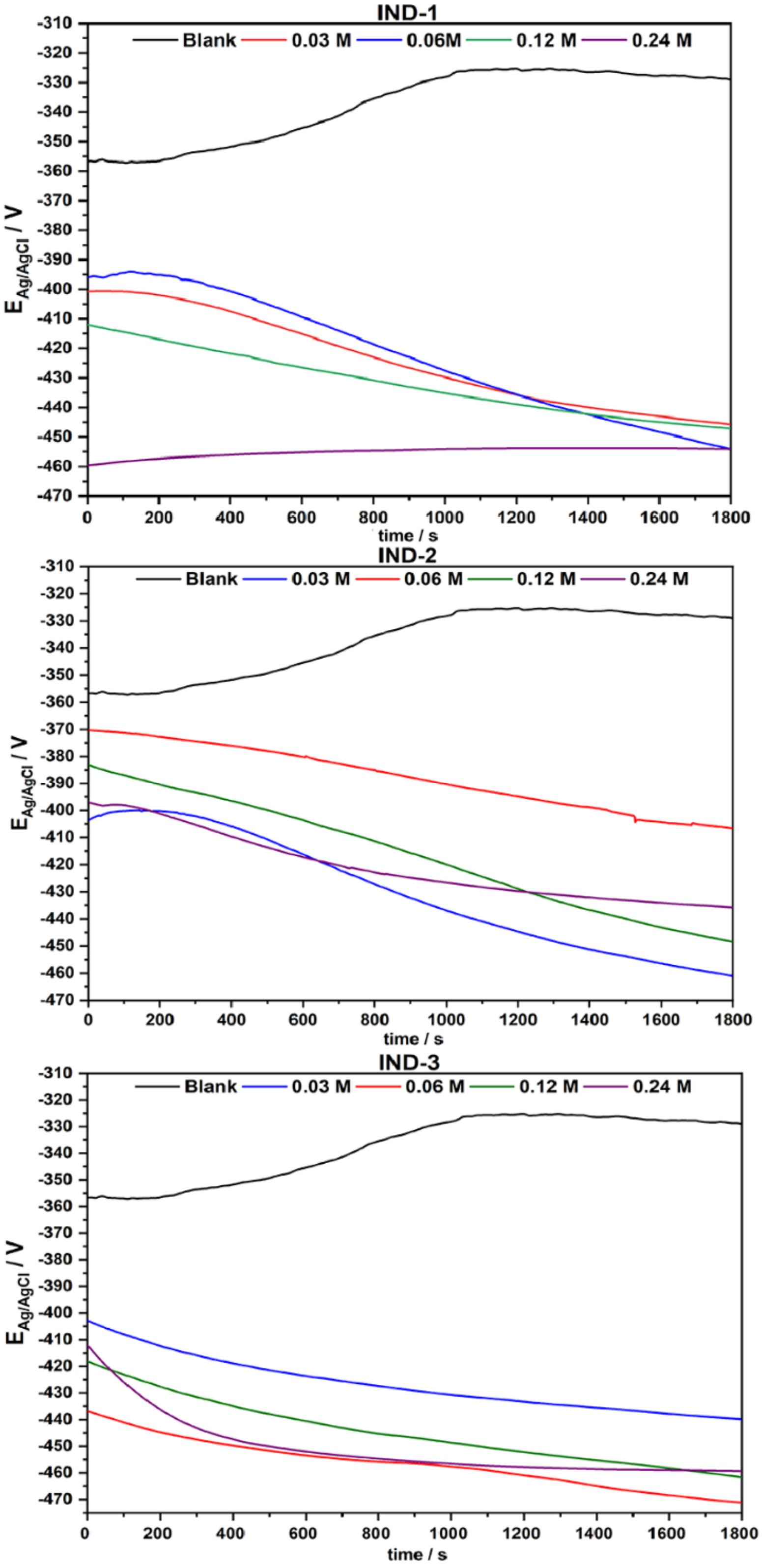

Figure S8. OCP vs. t of AISI 1018 in 1.0 M HCl of IND-1, IND-2, IND-3 at 298 K.

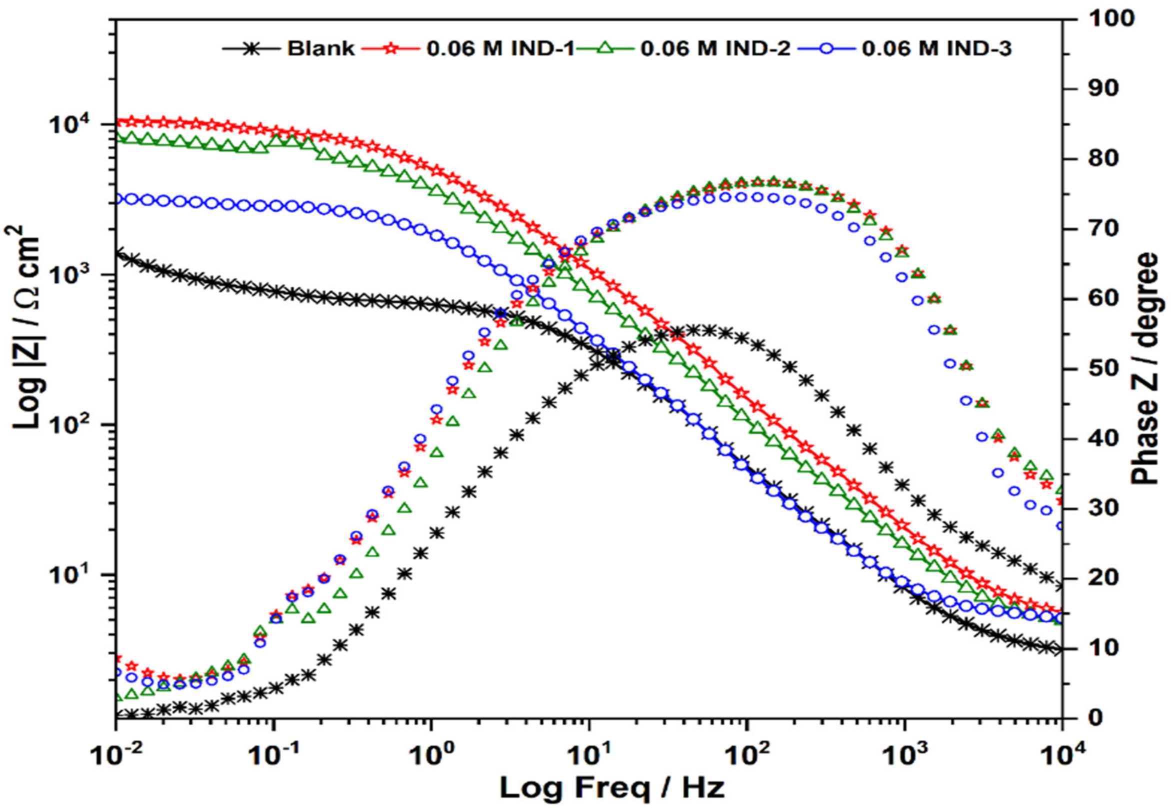

Figure S9. Bode Modulus and Bode Phase plots of the Blank (black line), IND-1 (red line), IND-2 (green line) and IND-3 (blue line) at 0.06 M.

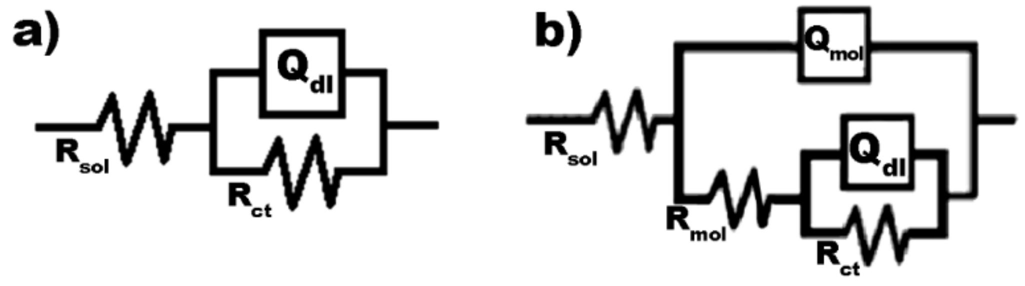

Figure S10. Equivalent electrical circuits were used to fit the experimental data for a) blank, b) with inhibitor

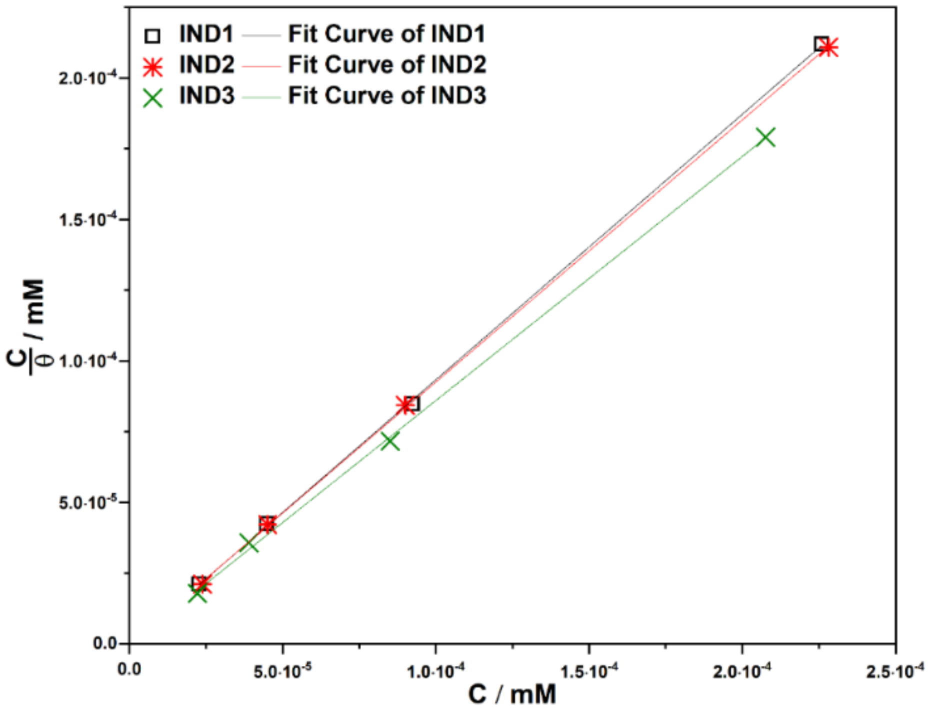

Figure S11. Langmuir Isotherm for the indanone compounds at 298 K.

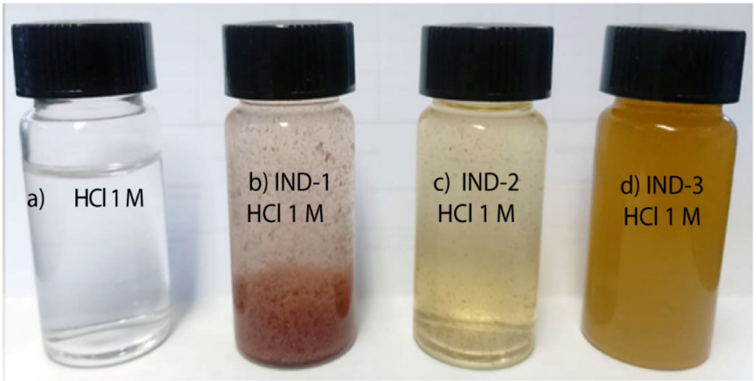

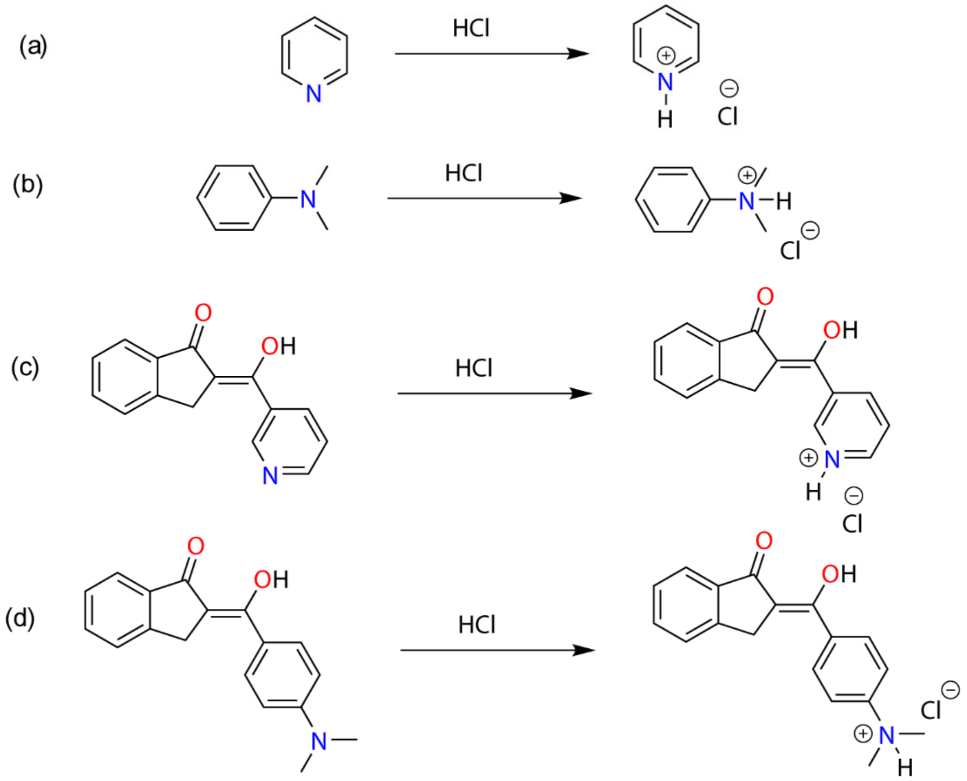

Figure S12. Photogram and Scheme of a) 1M HCl, b) IND-1 in 1M HCl, c) IND-2 in 1M HCl, and IND-3 in 1M HCl.

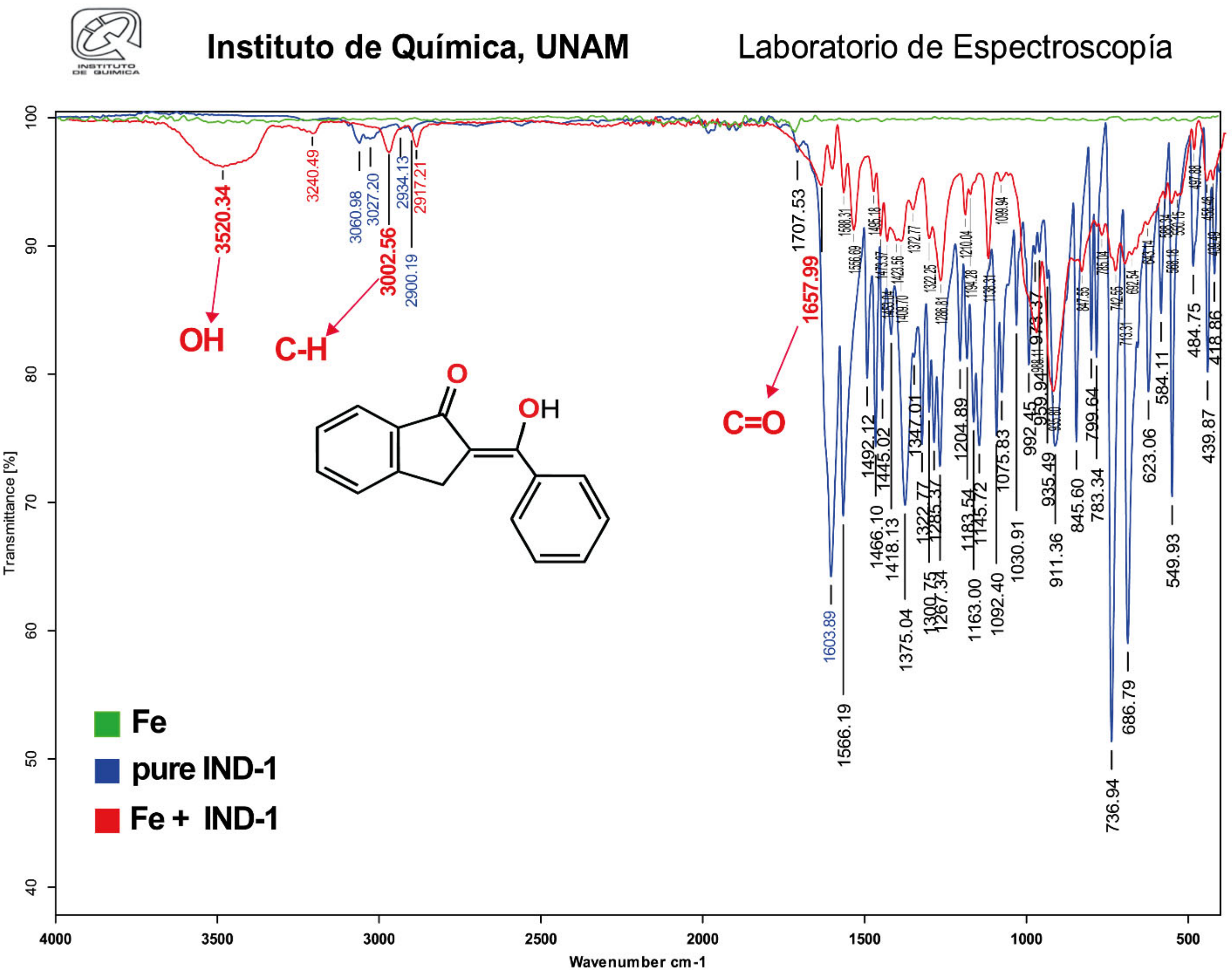

Figure S13. FTIR spectrum of Fe, pure IND-1 and Fe + IND-1

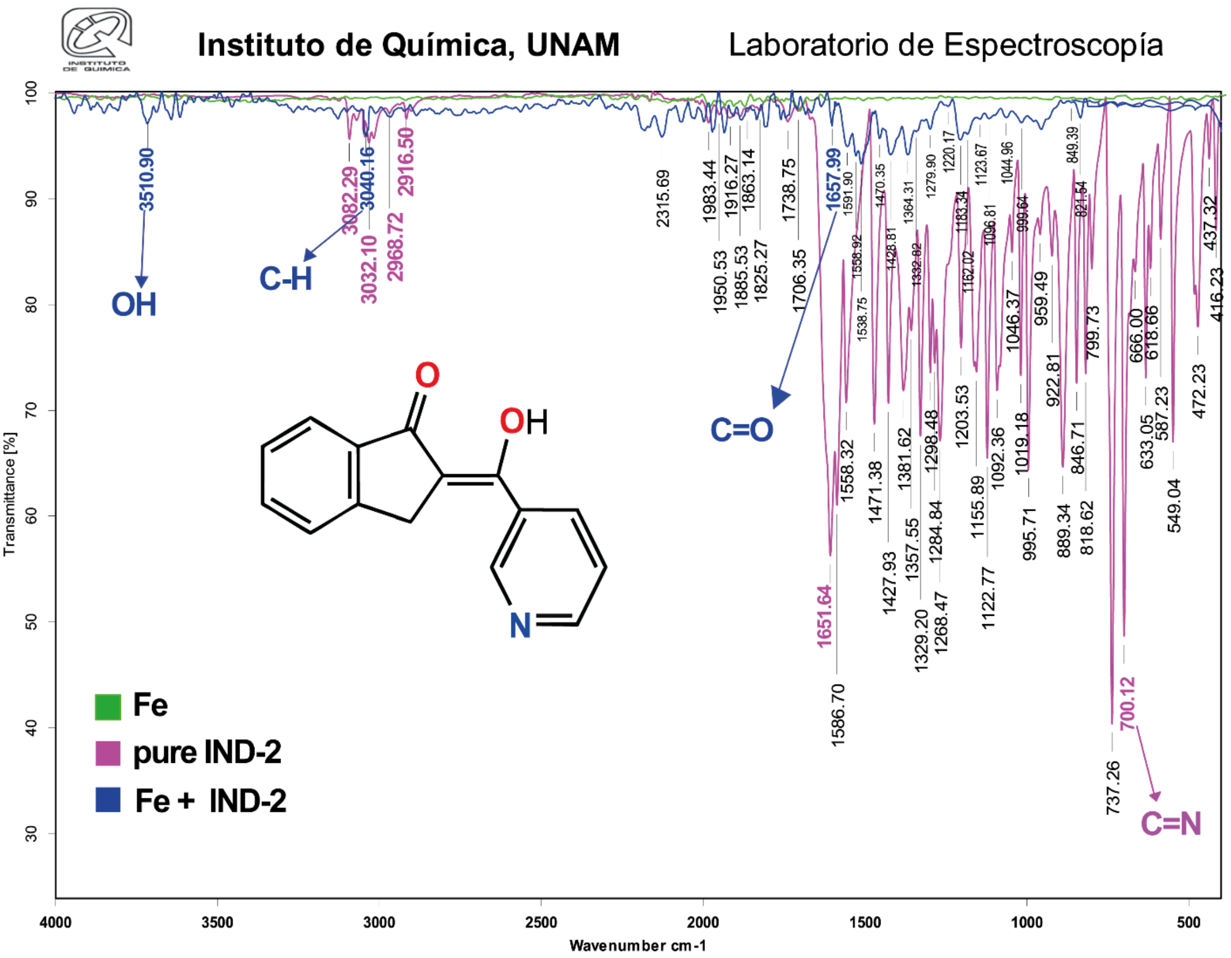

Figure S14. FTIR spectrum of Fe, pure IND-2 and Fe + IND-2

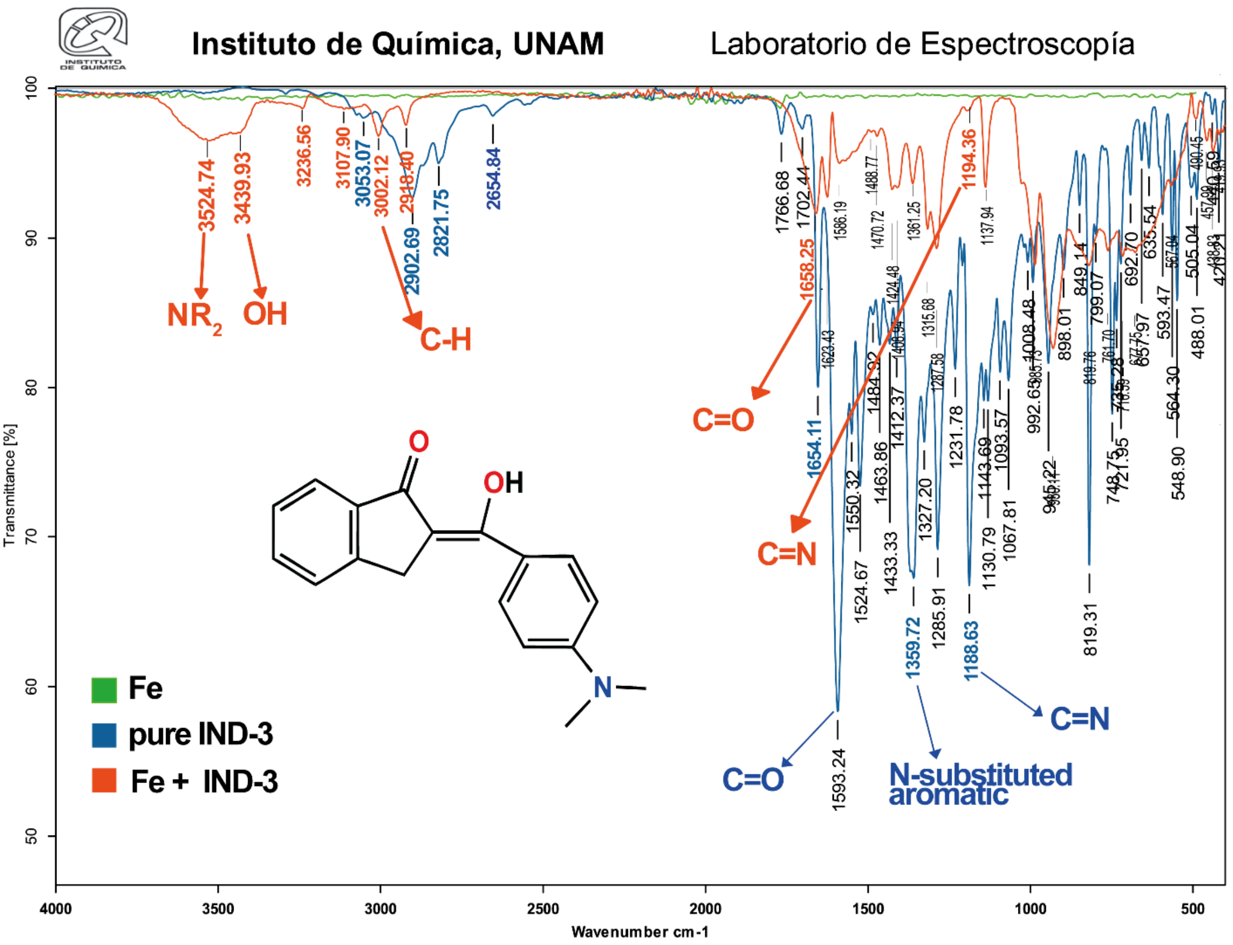

Figure S15. FTIR spectrum of Fe, pure IND-3 and Fe + IND-3

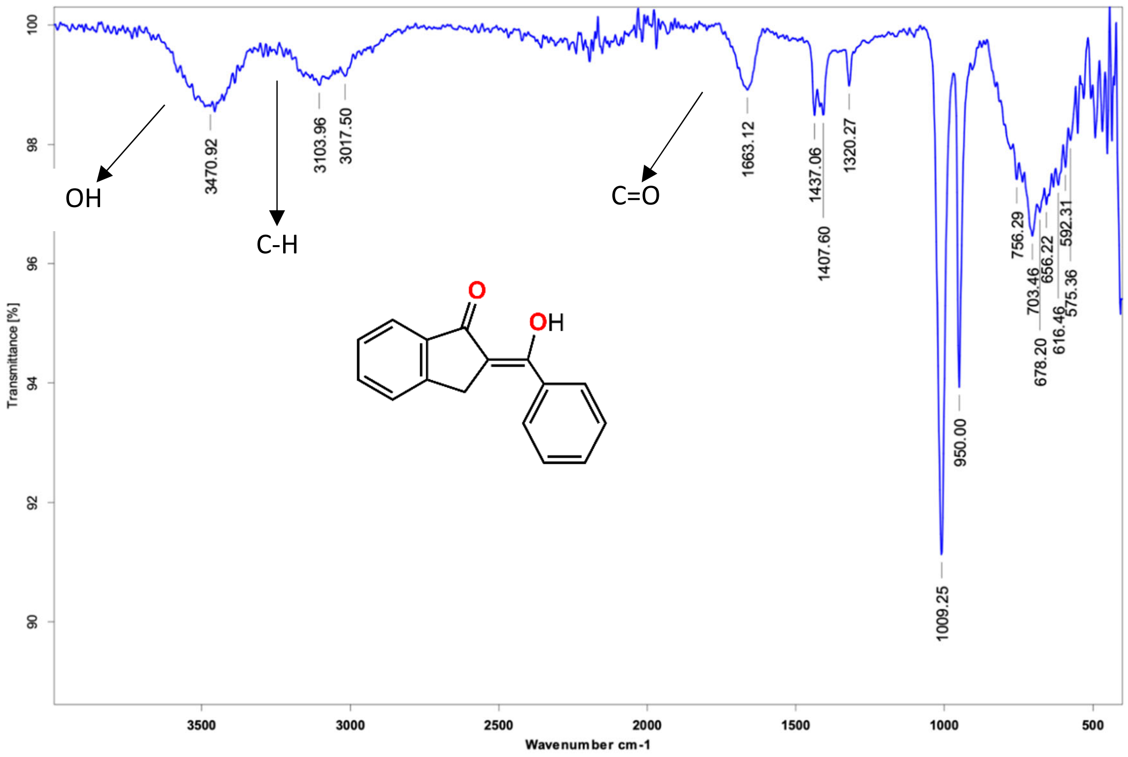

Figure S16. FTIR spectrum of IND-1 in 1M HCl.

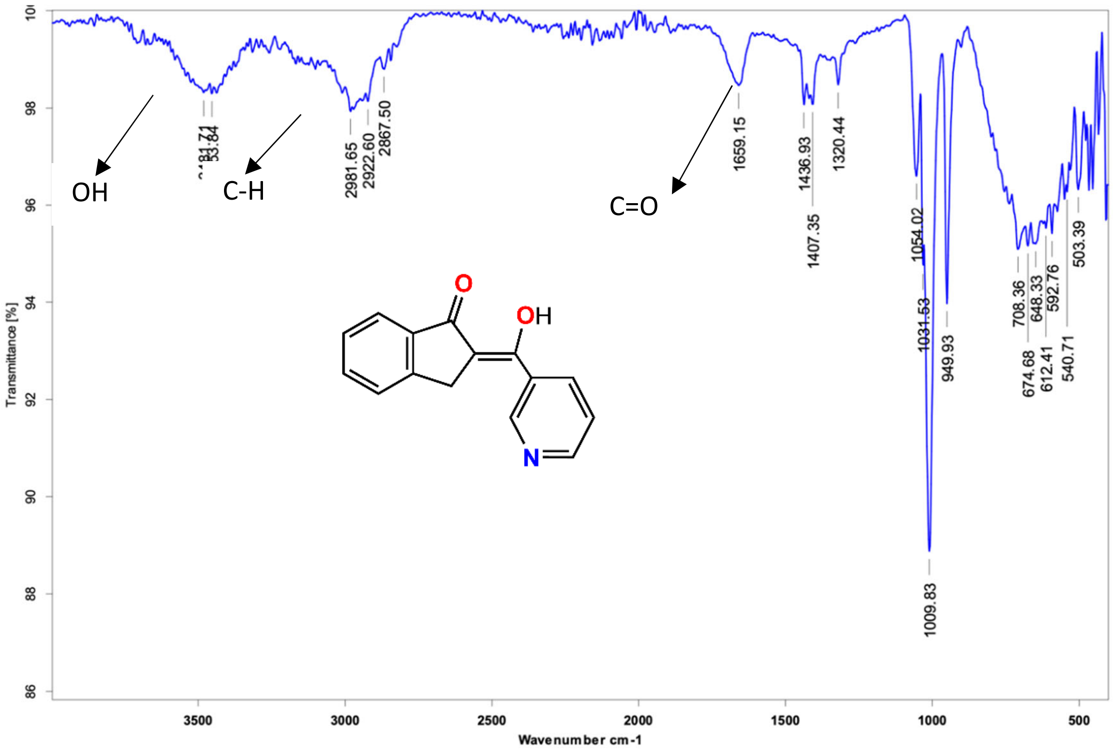

Figure S17. FTIR spectrum of IND-2 in 1M HCl.

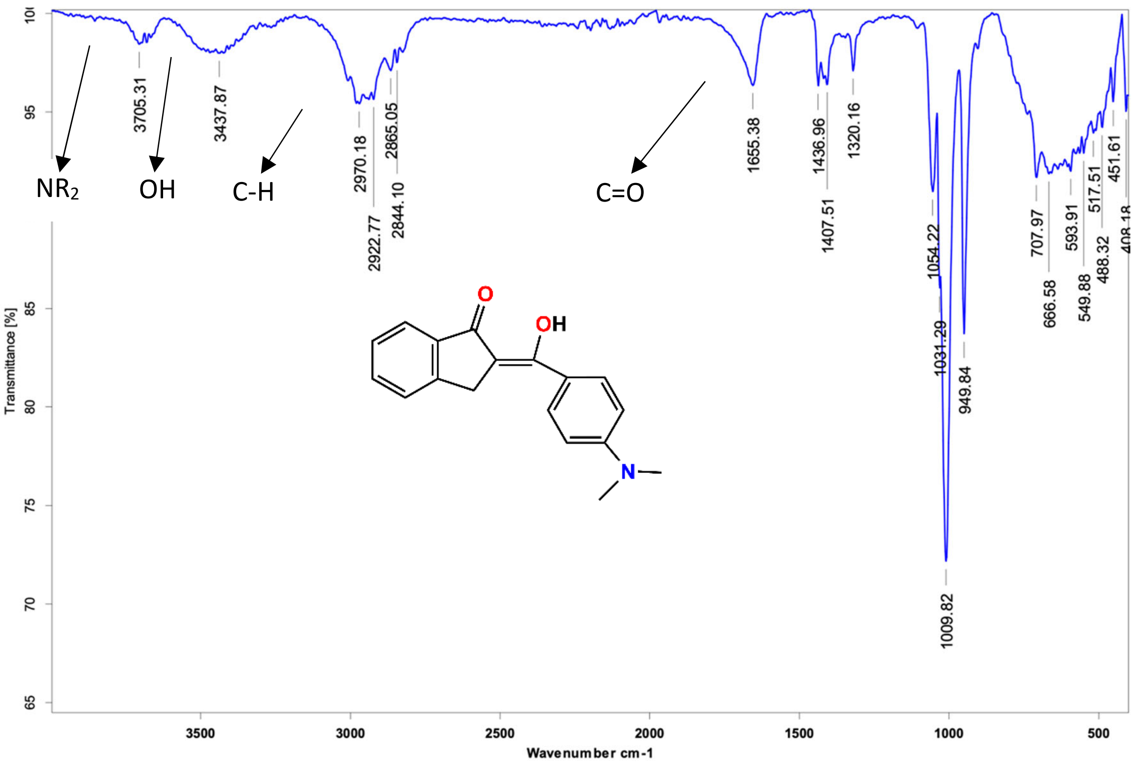

Figure S18. FTIR spectrum of IND-3 in 1M HCl.

TABLES

Table S1. Tafel extrapolation at IND-1, IND-2, and IND-3.

	Concentration / M	b_c /mV dec^{-1}	b_a / mV dec^{-1}	i_{corr} / µA cm^{-2}	CR_{corr} /mm year^{1}	η (%)
	0.0	-210.6 ±18.3	156.5 ±6.5	312.263 ±78.023	3.794 ±1.891	-
	0.03	-245.6 ±20.4	164.8 ±8.8	0.951 ±0.172	0.012±0.009	99.72 ±0.98
**IND-1**	0.06	-261.4 ±16.8	164.8 ±8.3	0.227 ±0.088	0.003 ±0.005	99.93 ±0.51
	0.12	-254.6 ±20.5	154.1 ±2.5	1.208 ±0.373	0.015 ±0.011	99.61 ±0.32
	0.25	-246.7 ±10.4	161.9 ±6.4	1.715 ±0.956	0.021 ±0.017	97.45 ±0.93
	0.03	-241.1 ±8.2	169.9 ±2.2	1.641 ±0.52	0.020 ±0.007	99.83 ±0.54
**IND-2**	0.06	-248.6 ±7.9	180.2 ±3.1	0.967 ±0.223	0.012 ±0.002	99.69 ±0.29
	0.12	-195.7 ±2.5	176.6 ±2.2	3.312 ±1.001	0.040 ±0.009	98.94 ±0.91
	0.25	-193.6 ±4.2	163.6 ±3.4	4.779 ±1.247	0.058 ±0.011	98.47 ±0.90
	0.03	-243.3 ±3.1	159.3 ±9.4	2.621 ±0.204	0.029 ±0.002	98.44 ±0.92
**IND-3**	0.06	-267.2 ±2.9	166.6 ±4.8	3.154 ±1.044	0.035 ±0.009	98.12 ±0.97
	0.12	-187.2 ±6.7	161.7 ±7.1	12.791 ±1.983	0.145 ±0.014	92.39 ±0.81
	0.25	-177.1 ±4.8	160.9 ±5.9	14.603 ±2.591	0.152 ±0.011	91.26 ±0.77

Table S2. Electrochemical parameters fit EEC for indanone derivatives.

C /M	R_{sol} / Ω cm^2	R_{mol} / Ω cm^2	Q_{mol} / μFcm^{-2}	$α_1$	R_{ct} /Ω cm^2	Q_{dl} / μFcm^{-2}	$α_2$	η /%
**IND-1**								
0.0	2.905±1.087	-	-		675.2 ±29.45	64.55 ±9.08	0.877	-
0.03	3.846 ±1.087	0.8119 ±0.278	29.35 ±2.54	0.785	9 866 ±53.816	14.73 ±1.65	0.965	93.07 ±0.93
0.06	3.571 ±1.113	61.277 ±2.921	31.07 ±5.08	0.718	13 483 ±70.145	7.01 ±2.71	0.995	94.46 ±0.32
0.12	4.679 ±1.187	51.550 ±3.045	7.884 ±3.22	0.757	10 115 ±92.489	26.38 ±7.07	0.986	93.76 ±1.06
0.25	5.908 ±1.472	58.451 ±4.191	7.111 ±2.92	0.704	8 978 ±30.478	32.26 ±8.27	0.973	91.68 ±0.87
**IND-2**								
0.03	1.426± 0.982	5.139 ±2.470	28.91 ±2.196	0.706	10 171 ±305.872	10.29 ±1.41	0.990	88.22 ±0.93
0.06	1.024± 1.214	6.347 ±2.087	28.45 ±1.096	0.765	11 370 ±147.342	9.97 ±3.47	0.993	93.66 ±0.51
0.12	2.049± 1.432	2.912 ±0.976	24.25 ±1.078	0.725	9 972 ±133.724	20.45 ±9.89	0.936	93.03 ±0.93
0.25	4.027± 1.774	0.325 ±0.212	29,77 ±1.769	0.751	9 112 ±116.578	46.52 ±3.04	0.997	92.76 ±0.93
**IND-3**								
0.03	5.388 ±1.335	13.278 ±0.376	7.99 ±2.76	0.707	5 446 ±201.243	36.77 ±2.17	0.959	87.60 ±1.78
0.06	4.045 ±1.376	16.331 ±0.170	22.36 ±1.96	0.717	7 122 ±190.047	20.37 ±1.97	0.904	91.69 ±0.99
0.12	3.606 ±1.184	3.707 ±0.221	49.75 ±5.27	0.721	3 862 ±97.488	40.82 ±2.11	0.927	76.41 ±1.91
0.25	5.956 ±1.472	3.942 ±0.087	10.10 ±4.08	0.723	2 016 ±50.316	65.20 ±4.73	0.898	66.84 ±1.79

Table S3. AFM data of carbon steel without inhibitor, IND-1, IND-2, and IND-3.

Sample	***Ra / nm***	***Rq /nm***	***R_{max} /nm***
	Before immersion		
Blank	18.755	24.291	142.5
	After immersion		
Blank	375.2	451.24	1798.6
IND-1	62.329	88.285	479.74
IND-2	113.37	147.75	899.06
IND-3	190.74	218.71	904.93

Table S4. Electronegativity of theoretical (eV) anticorrosion agents.

**Molecule**	**E_{HOMO} / eV**	**E_{LUMO} / eV**	**ω**
**IND-1**	-5.55	-1.77	3.78
**IND-2**	-6.12	-2.04	4.08
**IND-3**	-6.31	-2.26	4.52

CAPTION OF FIGURES & TABLES IN SUPPORTING INFORMATION

FIGURES' CAPTIONS

Figure S1. Synthesis of 2-benzylidene-1-indanone derivatives.
Figure S2. HRMS of IND-1.
Figure S3. ^{1}H-NMR spectrum of IND-1 in $CDCl_3$ 300 MHz.
Figure S4. HRMS of IND-2.
Figure S5. ^{1}H-NMR spectrum of IND-2 in CDCl3 300 MHz.
Figure S6. HRMS of IND-3.
Figure S7. ^{1}H-NMR spectrum of IND-3 in CDCl3 300 MHz.
Figure S8. OCP vs. t of AISI 1018 in 1.0 M HCl of IND-1, IND-2, IND-3 at 298 K.
Figure S9. Bode Modulus and Bode Phase plots of the Blank (black line), IND-1 (red line), IND-2 (green line) and IND-3 (blue line) at 0.06 M.
Figure S10. Equivalent electrical circuits were used to fit the experimental data.
Figure S11. Langmuir Isotherm for the indanone compounds at 298 K.
Figure S12. Photogram and Mechanism of a) 1M HCl, b) IND-1 in 1M HCl, c) IND-2 in 1M HCl, and IND-3 in 1M HCl.
Figure S13. FTIR spectrum of Fe (mild steel), pure IND-1 and steel of IND-1.
Figure S14. FTIR spectrum of Fe (mild steel), pure IND-2 and steel of IND-2.
Figure S15. FTIR spectrum of Fe (mild steel), pure IND-3 and steel of IND-3.
Figure S16. FTIR spectrum of IND-1 in 1M HCl solution.
Figure S17. FTIR spectrum of IND-2 in 1M HCl solution.
Figure S18. FTIR spectrum of IND-3 in 1M HCl solution.

TABLES' CAPTIONS

Table S1. Tafel extrapolation at IND-1, IND-2, and IND-3.

Table S2. Electrochemical parameters fit EEC for indanone derivatives.

Table S3. AFM data of carbon steel without inhibitor, IND-1, IND-2, and IND-3.

Table S4. Electronegativity of theoretical (eV) anticorrosion agents.
